# Academics’ Attitudes Toward Engaging in Public Discussions: Experimental Evidence on the Impact of Engagement Conditions

**DOI:** 10.1007/s11162-022-09725-4

**Published:** 2022-12-05

**Authors:** Vitus Püttmann, Jens Ruhose, Stephan L. Thomsen

**Affiliations:** 1 Institute of Economic Policy, Leibniz University Hannover, Königsworther Platz 1, 30167 Hannover, Germany; 2 Department of Economics, Kiel University, Olshausenstraße 40, 24098 Kiel, Germany; 3 CESifo Munich, Munich, Germany; 4 IZA Bonn, Bonn, Germany; 5 ZEW Mannheim, Mannheim, Germany

**Keywords:** Science communication, Public engagement, Professor, Survey experiment, Intrinsic motivation

## Abstract

Growing demands and expectations on the side of policy makers and the public have changed the conditions for academics’ engagement in public discussions. At the same time, risks related to this engagement for the professional and even private lives of academics have become apparent. Conducting a survey experiment among 4091 tenured professors in Germany, we study how these conditions causally affect academics’ attitudes toward engaging. Consistent with the crowding-out of intrinsic motivation, we find less-positive attitudes when emphasizing demands for engagement by public authorities and public expectations toward science’s societal relevance. Effects are particularly strong among professors endorsing science–society relations. Moreover, effects are similar when highlighting risks associated with engagement, but more pronounced for females and younger professors. Emphasizing public support for academics’ engagement has no discernible effects. We conclude that considering individual incentive structures and safeguarding against negative repercussions may promote academics’ engagement and an adequate representation of the diversity of academics in the public.

**Supplementary Information:**

The online version contains supplementary material available at 10.1007/s11162-022-09725-4.

## Introduction

In the wake of the challenges society has faced in recent years, academics have become an indispensable part of public discussions. Their appearances in the media and contributions to political debates and decision-making processes were pivotal when the COVID-19 pandemic unfolded—as the public prominence achieved by scientists like Anthony S. Fauci in the United States and Christian Drosten in Germany impressively shows. Similar efforts of academics have been and will continue to be essential for keeping climate change on the political agenda and ensuring that related debates take into account scientific evidence. A shared characteristic of these and other societal challenges is that their complexity and urgency make closer and more interactive relations between science and society necessary. Where longer processes of transferring academics’ expertise to the public such as via journalists in legacy media once sufficed, academics’ direct involvement in public debates has now become key.

As academics’ contribution to public discussions has become increasingly important, the conditions for it have changed in several respects. Policy makers have begun to pay greater attention to the academics’ engagement with the public and to devise policies, such as support initiatives for science communication activities, to promote it (Burchell, 2015; Mejlgaard, 2018; Weingart & Joubert, 2019). Similar expectations and support for academics’ activities in this area have been voiced by the public (Funk et al., 2019, 2020). In the United States, for instance, a public opinion poll conducted in 2019 found that 60% of respondents believed that scientists should be actively involved in policy debates related to scientific issues (Funk et al., 2019, p. 9). At the same time, risks associated with academics’ exposure to the public have become apparent (see, for instance, Nogrady, 2021). Two drastic examples of this are the cases of the German virologists Christian Drosten and Hendrik Streeck, who received death threats and were sued in relation to their public engagement during the COVID-19 pandemic (Zeit Online, 2020a, 2020b). Although these changes in conditions can be readily observed, their impact on academics remains an open question.

Academics’ willingness to engage with the public is strongly driven by their personal attitudes, presumably owing to a lack of integration of engagement activities into the academic profession. For example, scientists frequently attribute low priority to engagement activities (Rose et al., 2020; The Royal Society, 2006), and career benefits (both monetary and non-monetary) commonly trail other motives in terms of their perceived importance among those who do engage (Kreimer et al., 2011; The Royal Society, 2006; Torres-Albero et al., 2011). Whereas this suggests that engaging with the public is more a matter of personal conviction, there are signs that this might be changing. In addition to the importance ascribed to engagement by stakeholders of the science system and among the public, some academics do indeed report deriving career benefits from such engagement (Nisbet & Markowitz, 2015; Peters et al., 2008). In line with this ambiguous character of engagement activities, academics’ personal attitudes have been identified as a particularly relevant predictor of their engagement (Besley et al., 2013, 2018; Dudo, 2013; Poliakoff & Webb, 2007). This makes academics’ attitudes a useful object of inquiry when considering how changing conditions may influence their engagement behavior.

We investigate the influence of conditions on academics’ attitudes toward engagement in public discussions based on a survey experiment that we conducted as a randomized controlled trial (RCT) among 4091 tenured professors in Germany in late 2020. In the experiment, we ask respondents whether they would favor a reduction or an increase in academics’ engagement in public discussions in the future, and we examine how their answer behavior changes when we frame the question differently and provide selected pieces of information. For this, we randomly allocated respondents to either a control group receiving no further information and framing or one of four treatment groups, which covered the following conditions: external demands concerning academics’ engagement with society implicit in the legal framework (the *Legal duty* treatment); expectations concerning academics’ engagement among the public (the *Expectations public* treatment); public support for academics’ engagement with the public (the *Support public* treatment); and the risks associated with academics being exposed to the public (the *Examples risks* treatment).

Overall, we find high levels of support for an increase in academics’ engagement in public discussions. However, an emphasis on higher education institutions’ legal duties (*Legal duty* treatment) and on expectations among the public (*Expectations public* treatment) have a negative impact on these positive attitudes, especially for professors who have a positive stance on exchange relations between science and society. Less surprisingly, an emphasis on the risks associated with being exposed to the public (*Examples risks* treatment) also leads to less-positive attitudes, especially among younger and female professors. In contrast, an emphasis on support from the public (*Support public* treatment) does not have a discernible impact.

Our findings contribute to the scientific literature on relations between science and society, current science and higher education policy and management discussions, and the methodology of science and higher education research. First, previous research on the engagement of academics with the public has treated academics’ attitudes mainly as a predictor for engagement (for an exception, see Dudo, 2013). We show that these attitudes can also be investigated as an object of external influences and that engagement conditions currently in change are among the relevant influences. Second, the results of our study provide points of reference for science and higher education policy design and management. Especially at the policy level, recent attempts to promote academics’ engagement with nonacademic environments have often been embedded in a narrative referring to a change in expectations directed at science and to new duties of academics. Based on our findings, we question whether alluding to new expectations and duties is a sensible strategy. Our results suggest that a more promising strategy to foster relations between science and the public consists of devising institutional incentive structures that are in line with the intrinsic motivation of professors. Moreover, universities and public authorities should provide safeguards against the potential negative repercussions that can derive from academics’ exposure to the public. Third, our study highlights the usefulness and broader applicability of survey experiments. These have rarely been used for investigations covering academics thus far. Exceptions include primarily survey experiments using hypothetical scenarios such as curricula vitae (see, for instance, Carlsson et al., 2021; Ceci, 2018; see also Judson et al., 2019) or publication lists (see Powdthavee et al., 2018) that are varied systematically. Going beyond this approach, we show that using treatments inducing framing effects to gather insights into the relevance of specific considerations works with highly educated individuals in scientific fields.

## Background: Academics’ Public Engagement

### Changing Conditions

Since the second half of the twentieth century, policy discussions have paid increasing attention to academics’ communication and, later on, direct engagement with broader audiences. These activities have found their way into science and higher education policy agendas in many countries (Burchell, 2015; Chikoore et al., 2016; Mejlgaard, 2018; Mejlgaard et al., 2012; Torres-Albero et al., 2011; Weingart & Joubert, 2019). Differences among national contexts notwithstanding, this reality is evident in the relevance ascribed to science communication and engagement in political strategizing, the adjustment of research funding mechanisms, and the establishment of numerous support initiatives. These efforts of governments are in many cases reinforced by those of other stakeholders, such as learned societies and foundations.

The orientation in the policy sphere is complemented by similar sentiments within societies more generally. Overall trust in science remains high or has even increased in many countries (Funk et al., 2020). Moreover, significant portions of the population expect science to contribute to societal development and show support for academics’ engagement in public debates (BEIS, 2020; Funk et al., 2019; Wissenschaft im Dialog, 2019). In Germany, for instance, 67% of respondents to a 2019 public opinion poll agreed with the statement that the expected contributions to solving societal problems should guide decisions regarding science and research (Wissenschaft im Dialog, 2019, p. 30). A public opinion poll conducted in the same year in the United States revealed that 60% of respondents believed that scientists should be actively involved in policy debates related to scientific issues (Funk et al., 2019, p. 9).

As demands and expectations directed at academics have intensified, challenges associated with their public engagement have become increasingly visible. The logics and dynamics of public discussions differ and can even contradict those of communication within the scientific community. These differences have been amplified by the diversification of communication channels. The expansion of online media, and social media in particular, has ended the position of legacy media—such as print media and television—as the main link between science and public discourse. In addition to science being covered in online media, academics themselves, especially younger scholars (Besley et al., 2018), are using online channels of communication. In blogs, microblogs, and social networks, they communicate research results to audiences outside of the scientific community and engage in public discussions (Jensen, 2011; Hamlyn et al., 2015; Jünger & Fähnrich, 2020; see also Sugimoto et al., 2017). These new channels enhance the opportunities academics have to communicate and directly interact with broader audiences and provide additional possibilities for immediate feedback. This, however, implies that those who engage can be confronted with hostility in debates concerning contentious and polarizing issues. As a result, there is a constant risk that academics might experience negative repercussions that can extend to their professional and even private lives (see, for instance, Nogrady, 2021).

### Individual Perspective

From the perspective of academics themselves, engaging with audiences outside of the scientific community is different from other academic activities. On the one hand, it appears that this engagement, in its various forms, is more a matter of personal conviction than a genuine part of the academic profession. The perception that it is accorded a low priority, including by academic peers, is common (see Rose et al., 2020; see also Hamlyn et al., 2015; The Royal Society, 2006). This view corresponds to the motives academics in various countries report having for engagement (Kreimer et al., 2011; Peters et al., 2008; The Royal Society, 2006; Torres-Albero et al., 2011; see also Burchell, 2015; Poliakoff & Webb, 2007). Motives related to the standing of science in society, such as informing the public and improving its perspective on science, are usually considered more relevant than those related to academic career advancement. On the other hand, there are signs that public engagement activities have become more accepted as genuinely academic activities. Another important motive frequently reported by academics is that of fulfilling a perceived duty. Moreover, personal career benefits have been found to be relevant in some studies, especially with regard to academics’ engagement via the mass media (Besley & Nisbet, 2013; Nisbet & Markowitz, 2015; Peters et al., 2008).

In line with this peculiar role, academics’ personal attitudes toward engaging with the public have been identified as one of the most important and persistent predictors of their engagement (Besley et al., 2013; Besley et al., 2018; Poliakoff & Webb, 2007; Dudo, 2013; see also Dunwoody et al., 2009). For example, Besley et al. (2018) use survey data collected in 2015 and 2016 on 4703 natural scientists in the United States to show that the perceived impact and, in particular, scientists’ general attitude toward engagement are the most relevant predictors of their willingness to engage. Furthermore, the effects these authors observe hold across the three modes of engagement considered, namely, face-to-face communication, communication via the media and communication via online channels.

Despite their ability to predict engagement, academics’ attitudes have rarely been investigated as subject to external influences. A few studies conjecture that these attitudes may be shaped by other beliefs (Besley et al., 2013; Dudo, 2013), norms (Poliakoff & Webb, 2007) or factors such as media consumption (Dudo, 2013) and may thereby act as a mediator for effects on academics’ willingness to engage. Based on a survey of 363 biomedical researchers in the United States, Dudo (2013) indeed finds evidence for such a mediating effect for (see also Besley et al., 2020), among others, the consumption of print media. However, the extent to which academics’ attitudes are open to external influences and which factors are relevant in this regard have not been investigated systematically thus far.

## Research Design and Data

### Experimental Design

**Fig. 1:**
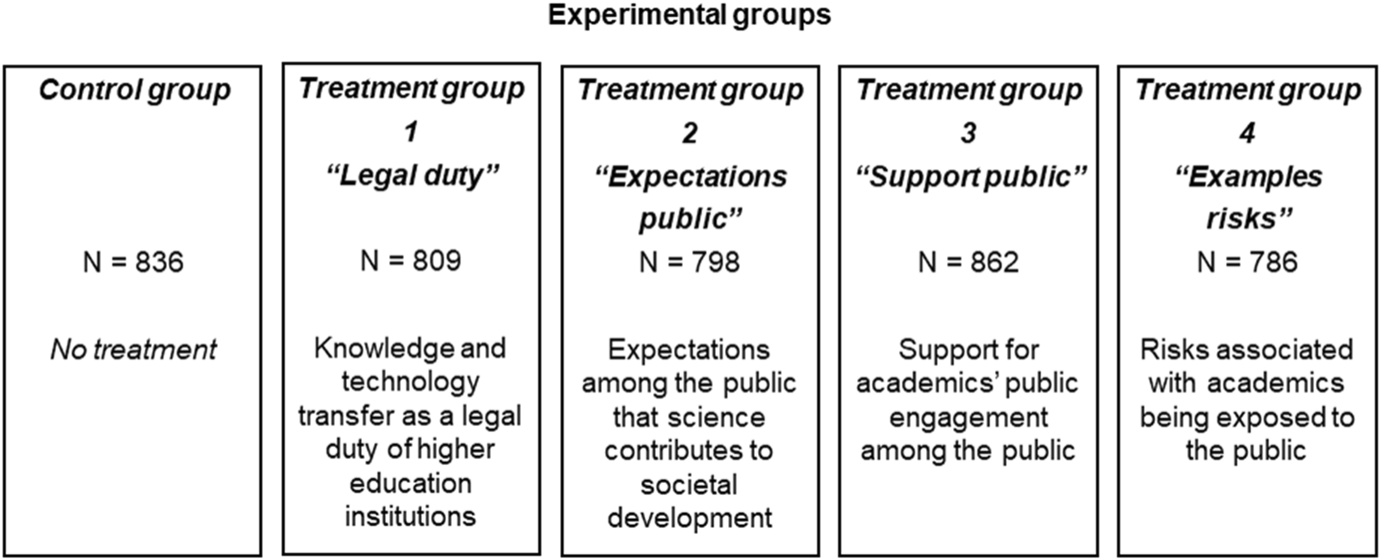
Experimental groups. *Notes* The figure shows the experimental groups. Online Appendix Fig. A.1 shows the design of the survey experiment; Online Appendix Fig. A.2 shows how the experiment was embedded in the overall survey; Online Appendix Fig. A.3 shows the original German version

To investigate the impact of conditions on academics’ attitudes toward engaging in public discussions, we conducted a survey experiment as an RCT with four treatment groups and one control group through an online survey of professors in Germany (see Fig. 1 for an overview of the experimental groups and Online Appendix Fig. A.1 for an overview of the experimental design).^1^ The experiment consisted of two questions eliciting all respondents’ prior knowledge of the information provided by three of the four treatments and one question, into which the four treatments are embedded, producing the experiment’s outcome. The survey comprising the experiment addressed the relation of respondents with the nonacademic environment (see Online Appendix Fig. A.2 for an overview of the structure of the survey). The main topics included respondents’ perspective on the relations between science and society and their own involvement in activities in the areas of knowledge and technology transfer, continuing education and societal engagement. A section toward the end of the survey was dedicated to the impact of the COVID-19 pandemic on the respondents and comprised the experiment.

The outcome of the experiment consisted of an indicator of respondents’ attitudes toward engagement in public discussions. Following an introductory sentence transitioning from the topic of the COVID-19 pandemic and stating that *the relevance of science and scientific findings for public opinion formation and political decisions has also increased outside the context of crises*, all respondents were asked the following question: *If you consider academics from your own discipline, should these academics become less or more involved in public discussions in the future?* Responses were given on a 5-point scale ranging from *much less* to *much more* (with *the same amount* as the mid-point). One randomly selected group of respondents received only this question and serves as the control group. The four other randomly selected groups received altered framings of the question and additional pieces of information as treatments (for the exact wording in German, see Online Appendix Fig. A.3).

The first treatment—the *Legal duty* treatment—focused on the uptake of science–society relations in the legal framework for higher education and the demands directed at academics that at least implicitly derive from this. Citing a recent research article (namely, Berghäuser, 2017), the treatment informed respondents that in addition to the general relevance of science for the public, *legislators have included knowledge and technology transfer as a general duty of higher education institutions in the higher education laws of all German federal states except for Hamburg*. This treatment might induce more positive attitudes if academics perceive the demands as a signal that engaging in public discussions has become a valued academic activity. However, if the demands are perceived as intruding on academics’ professional autonomy, they might just as easily induce more negative attitudes.

The second treatment—the *Expectations public* treatment—referenced expectations within the population concerning the relation between science and society. It reframed the question by pointing out that the general relevance of science for the public *is also mirrored in the German population’s expectations of science*. It then informed respondents that *in a poll conducted in 2019, 67*% *of respondents stated that they agree with the following statement: “Decisions on science and research should primarily be based on their contribution to solving societal problems”*; the source of the data was also mentioned (i.e., Wissenschaft im Dialog, 2019). This treatment might also induce either more positive or more negative attitudes depending on whether the public’s expectations are perceived as a reassurance of the value attributed to academics’ engagement or as an infringement on academics’ autonomy.

The third treatment—the *Support public* treatment—referenced public support for academics’ engagement in public discussions. It reframed the question by pointing out that in addition to the general relevance of science for the public, *there is broad support among the German population for academics’ public engagement*. It then referenced the same informational source (i.e., Wissenschaft im Dialog, 2019), stating that *in a poll conducted in 2019, 75*% *of respondents stated that they agree with the following statement: “Academics are right to speak out in public when political decisions do not take research results into account”*. If anything, we would expect this treatment to shift academics’ attitudes in a more positive direction.

The fourth treatment—the *Examples risks* treatment—covered the risks associated with academics’ engagement in public discussions. It reframed the question by pointing out that *the public engagement of academics also entails risks*. It then provided two examples of these risks, referring to two scientists who played a prominent role during the COVID-19 pandemic in Germany comparable to the role Anthony S. Fauci played in the United States. It stated that *Professor Christian Drosten, Director of the Institute of Virology at the Charité Berlin, reported receiving death threats in relation to his public appearance in the context of the COVID-19 pandemic* and that *Professor Hendrik Streeck, Director of the Institute of Virology at the University Hospital Bonn, was sued in relation to a study on the COVID-19 pandemic*. Two newspaper articles (i.e., Zeit Online, 2020a, 2020b) were provided as the sources of this information. Aside from physical violence, death threats are among the most negative repercussions that an individual can face. We therefore interpret this treatment as a test of the upper negative bound that can be expected from emphasizing the risks associated with academics’ engagement in public discussions.

By providing a specific framing of the question as well as selected pieces of information, the treatments can influence the outcome via two mechanisms. First, via the framing provided, they increase the salience of certain considerations concerning academics’ engagement in public discussions among respondents during the response process (for a discussion of framing effects, see Chong & Druckman, 2007; see also Zaller & Feldman, 1992). Second, via the information provided, they might correct respondents’ prior knowledge about the issue referred to by the treatment. If the issue covered by the treatment is relevant to the respondent, both mechanisms could alter the opinions expressed. To be able to discern the two mechanisms, we consider in further analysis the respondents’ prior knowledge about the quantifiable information included in the first three treatments.

### Data Collection

The target population of the survey included all professors at German higher education institutions governed by the state (except for civil service institutions) or by religious institutions. We identified 45,635 individuals belonging to the target population based on two sources. For professors at universities and some art and music colleges, we used the online version of the *Hochschullehrer Verzeichnis 2019* (DHV, 2019), a regularly updated register of professors edited by the German Association of University Professors and Lecturers (*Deutscher Hochschulverband*). For professors at institutions not covered by this register, we reverted to institutions’ websites. No adequate contact details could be obtained for 502 professors, and 501 professors were included in a pretest of the survey questionnaire,^2^ leading to a gross sample of 44,632 individuals.

The survey was distributed online between October 5 and November 15, 2020. All individuals in the gross sample received an invitation via e-mail or via an online contact form. Those who had not yet completed the survey received a reminder 8 and 22 days after the initial invitation. In 1844 cases, all three contact attempts failed due to, among other reasons, inactive e-mail addresses or spam filters. An additional 703 cases were identified as not belonging to our target population based on feedback during the field phase and the survey results. This led to an adjusted gross sample of 42,085 individuals.

Overall, the survey yielded 4726 valid responses, amounting to a net response rate of 11.2%. This response rate is similar to that of two recent Germany-wide scientific surveys by the *German Centre for Higher Education Research and Science Studies* (DZHW). Those surveys obtained response rates from professors, excluding those at universities of applied sciences, of 10.0% in 2016 (Neufeld & Johann, 2018) and 12.4% in 2019/2020 (Ambrasat et al., 2020). For the purpose of this analysis, we restricted the sample to the 4173 tenured professors. That is, we kept associate and full professors (salary grades W2/C3 and W3/C4) and dropped from the analysis assistant professors (salary grade W1/C2), professors with fixed-term contracts and respondents who did not specify the type of professorship. Tenured professors possess a high level of autonomy in regard to deciding which activities to engage in. This should allow us to observe the effects of the treatments with as few confounding influences as possible. Out of this group, 80 observations were removed from the analysis because of missing responses to the question forming the experiment’s outcome. We checked the remaining observations for sufficient data quality, speeding, and straightlining. None of these observations exhibited a share of missing values for the main survey items above 40% or a response time below one-third of the median. A check of the survey’s six main item batteries for straightlining revealed that the response pattern varied across at least half of the batteries in all except two cases, which were therefore excluded from the analysis. Thus, our analytical sample contains 4091 observations.

### Variables

The experiment’s outcome is transformed from the original 5-point scale format, as the distribution of the outcome variable is concentrated at the upper end of the scale. In the control group, 51.6% of respondents support more engagement, and an additional 23.8% support much more engagement (see Table 1). The outcome therefore enters the analysis in two forms: as a dummy variable indicating support in general (i.e., those advocating more or much more engagement) and as a dummy variable indicating strong support (i.e., exclusively those advocating much more engagement). This allows us to distinguish between two potentially complementary shifts in respondents’ attitudes: first, a general shift toward or away from supporting an increase in engagement (“general support”), and, second, a shift that primarily concerns the strength with which respondents support an increase in engagement (“strong support”).

**Table 1:** Descriptive statistics for the sample

	Sample		Population		Chi-squared test	
	*N*	*%*	*N*	*%*	*χ*^*2*^	*p*-value
	(1)	(2)	(3)	(4)	(5)	(6)
Preferred extent of engagement in public discussions (control group only)					–	–
Much less	6	0.7	–	–		
Less	20	2.4	–	–		
The same amount	180	21.5	–	–		
More	431	51.6	–	–		
Much more	199	23.8	–	–		
Gender					1.176	0.278
Male	2,985	75.2	29,470	75.9		
Female	983	24.8	9,345	24.1		
Age group					–	–
< 45 years	603	15.1	–	–		
45–54 years	1,565	39.1	–	–		
> 54 years	1,833	45.8	–	–		
Type of professorship					31.221	0.000
Associate professor	2,626	64.2	23,345	60.1		
Full professor	1,465	35.8	15,470	39.9		
Group of academic disciplines					51.998	0.000
Engineering sciences	1,243	30.6	11,015	28.8		
Law, economics, social sciences and sports science	1,160	28.5	10,175	26.6		
Humanities and arts	696	17.1	6,885	18.0		
Mathematics and natural sciences	635	15.6	5,870	15.3		
Medicine, health sciences and others	334	8.2	4,325	11.3		
Type of institution					171.284	0.000
University	1,841	46.3	21,675	55.8		
University of applied sciences	1,930	48.5	15,215	39.2		
College of art/music	207	5.2	1,925	5.0		
Region					4.021	0.045
West Germany	3,156	79.4	31,265	80.5		
East Germany	821	20.6	7,550	19.5		

The table shows the absolute number and share of professors in the sample and, where publicly available, the population by selected characteristics (for details see “Variables” section), and the results of Pearson's chi-squared tests. For the preferred extent of engagement in public discussions, data on the sample cover exclusively the control group. Data on the population are based on official higher education statistics and were retrieved via the information system *DZHW ICEland* (data set 60102). In contrast to the sample, data for the population include professors without permanent contracts. Totals among characteristics differ due to differences in categorization between the survey and the secondary data as well as item non-response in the survey data. “others” includes agronomy, forestry, nutrition science and veterinary medicine

We add three sets of covariates as control variables. These include information on respondents’ engagement with the nonacademic environment, basic features of their academic employment, and demographic characteristics. Accounting for basic differences between these groups allows us to increase the precision of our estimates. We furthermore consider respondents’ personal characteristics a potential source of effect heterogeneity and thus analyze the effects of our treatments separately for subgroups based on the demographic, occupational, and engagement characteristics.

Information on respondents’ engagement with the nonacademic environment covers their attitudes and actual engagement. Two variables cover the importance that respondents ascribe to knowledge and technology transfer and to societal engagement as part of a professorship from their personal perspective. These assessments were transformed from a 5-point scale to two groups, contrasting those who consider these activities *not at all*, *not that* or only *somewhat important* with those who consider them *very* or *extremely important*. A second set includes three dummy variables designating whether respondents used the following three channels for communicating scientific findings to audiences outside of the scientific community in 2019: press, radio or television; lectures or panel discussions; and online social networks.

As basic features of respondents’ academic employment, we include the type of professorship (associate professor, i.e., those at the W2/C3 salary grade; full professor, i.e., those at the W3/C4 salary grade) and five groups of academic disciplines: engineering sciences; law, economics, social sciences and sports science; humanities and arts; mathematics and natural sciences; and medicine, health sciences, veterinary medicine, agronomy, forestry and nutrition science. We also add the type of institution (university; university of applied sciences; college of art/music) and its location in West Germany or East Germany. As demographic characteristics, we include gender (male; female^3^) and the age group (up to 44 years; 45 to 54 years; 55 years or older) of respondents.

### Descriptive Statistics

Our analytical sample shows a rather positive stance on the preferred extent of academics’ engagement in public discussions (see Table 1). In the control group, which serves as the benchmark in the following analysis, only 3.1% of respondents advocate a reduction in engagement, and only 21.5% do not see a need for changing the current level. Instead, 51.6% are in favor of *more* engagement and 23.8% are even in favor of *much more* engagement.

Different groups of professors are well represented in our sample (see Table 1 and Online Appendix Table B.1), even though the distribution in the sample differs from that in the population in some cases. Due to the comprehensiveness of the survey, the sample comprises professors from more than 240 higher education institutions, professors from all academic disciplines, professors from all age groups and professors with different personal views on science–society relations. The most pronounced difference between the sample and the population is an underrepresentation of professors employed at a university (by 9.5 percentage points) and an overrepresentation of professors employed at a university of applied sciences (by 9.3 percentage points). One reason for this could be the stronger application and transfer orientation at universities of applied sciences, which made our survey more relevant to professors at this type of institution.

While we cannot rule out that the openness to the nonacademic environment exhibited by the professors in our sample could also be partly due to the questions about the extent of public engagement presented before, we provide some evidence that the answer behavior is likely a result of self-selection into the survey. To gauge the extent of self-selection, we compared the levels of engagement with actors outside of the scientific community in our sample to those found in another survey with a similar target group. As part of the *Academic Profession in Knowledge Societies* (APIKS) research project (Schneijderberg & Götze, 2020), academics in Germany were surveyed about their general employment and working conditions. Given this thematic focus, the results of the APIKS survey should provide us with a suitable reference point for assessing the particularities of our sample in terms of professors’ engagement with the nonacademic environment. Even though differences in sample composition and measurement pose challenges for a direct comparison, we do observe markedly higher shares of professors engaged in contract research and consulting and in exchanges with the public in our sample than that found in the one of the APIKS survey (see Online Appendix Table A.1). In addition, the questions covering the importance respondents ascribe to knowledge and technology transfer and societal engagement were located at the very beginning of the survey and should therefore be less affected by demand effects. The fact that the responses to these questions (see Online Appendix Table B.1) conform to the general picture concerning professors’ openness to the nonacademic environment also suggests that self-selection into the survey may well explain respondents’ openness.

The generally positive stance on exchange relations with society and the comparatively high levels of engagement with the nonacademic environment in particular (see Online Appendix Table B.1) suggest that professors with negative views on engagement in public discussions are underrepresented in our sample. Because randomization into the treatment groups occurred after self-selection into the survey, the internal validity of our estimates presented below is not affected. However, the selected sample creates potential issues for the external validity of the results. Arguably, the sample covers well the population that is of interest for our analysis because we do not believe that our treatments are so powerful that they can change the minds of those who are staunchly opposed to public engagement activities.^4^ We nevertheless conduct further analyses to assess the relevance of our sample composition: In Online Appendix C, we replicate our main estimations using survey weights based on data for the population (which does not alter our findings in any meaningful way). Moreover, in the “Effect Heterogeneity” section, we examine the treatment effects for subgroups in our sample.

## Empirical Results

### Main Treatment Effects

**Fig. 2:**
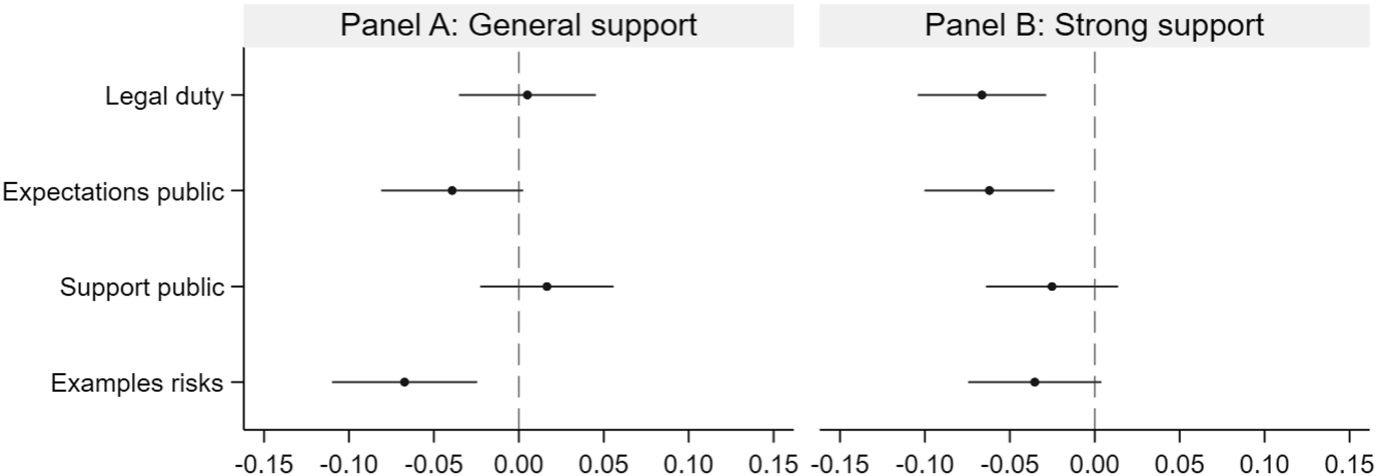
Main effects of the treatments. *Notes* The figure shows the point estimates and the 95% confidence intervals of the treatment indicators estimated from linear probability models (ordinary least-squares regressions with robust standard errors) regressing the dummy variable indicating respondents’ (strong) support for an increase in engagement on the treatment indicators. Full sets of demographic, occupational, and engagement characteristics are always included. See “Variables” section and Online Appendix Table B.1 for details. Missing values for the control variables are imputed, and imputation dummies are included in the regressions. The point estimates represent the change in the probability of (strongly) supporting an increase in academics’ engagement in public discussions due to receipt of the treatment. The average in the control group is 0.754 for the dummy variable for general support and 0.238 for the dummy variable for strong support. Number of observations = 4091. The coefficient plot was produced using the Stata module *coefplot* (Jann, 2014)

In the main analysis, we investigate whether the four treatments (see Fig. 1 for an overview) influence the probability that respondents support an increase in academics’ engagement in public discussions.^5^ We measure the treatment effects by estimating linear probability models regressing each of the two outcome variables, i.e., the indicators for general support and strong support, on the treatment indicators and the control variables using the full sample.The main results are presented in Table 2 and Fig. 2. Panel A in both the table and the figure shows whether the respondent *generally* supports an increase in public engagement by professors. Panel B refers to the second outcome variable, which measures whether the respondent *strongly* supports an increase. The inclusion of the control variables covering demographic, occupational and engagement characteristics in the estimations increases the precision of our estimates.

**Table 2:** Main effects of the treatments

	(1)	(2)	(3)	(4)
Panel A: General support				
Legal duty	0.007 (0.021)	0.010 (0.021)	0.009 (0.021)	0.005 (0.021)
Expectations public	− 0.032 (0.022)	− 0.032 (0.022)	− 0.033 (0.022)	− 0.039* (0.021)
Support public	0.024 (0.021)	0.024 (0.020)	0.025 (0.021)	0.017 (0.020)
Examples risks	− 0.068*** (0.022)	− 0.068*** (0.022)	− 0.068*** (0.022)	− 0.067*** (0.022)
Observations	4,091	4,091	4,091	4,091
R-squared	0.005	0.012	0.018	0.061
Panel B: Strong support				
Legal duty	− 0.065*** (0.020)	− 0.064*** (0.020)	− 0.064*** (0.020)	− 0.066*** (0.019)
Expectations public	− 0.058*** (0.020)	− 0.056*** (0.020)	− 0.057*** (0.020)	− 0.062*** (0.020)
Support public	− 0.020 (0.020)	− 0.020 (0.020)	− 0.020 (0.020)	− 0.025 (0.020)
Examples risks	− 0.040* (0.020)	− 0.039* (0.020)	− 0.038* (0.020)	− 0.035* (0.020)
Observations	4,091	4,091	4,091	4,091
R-squared	0.004	0.008	0.015	0.058
Demographic characteristics		x	x	x
Occupational characteristics			x	x
Engagement characteristics				x

The table shows the coefficients and standard errors of the treatment indicators estimated from linear probability models (ordinary least-squares regressions with robust standard errors) regressing the dummy variable indicating respondents’ (strong) support for an increase in engagement on the treatment indicators. *Demographic characteristics:* gender (2 categories) and age (3 categories). *Occupational characteristics:* type of professorship (2 categories), group of academic disciplines (5 categories), type of institution (3 categories), and region (2 categories). *Engagement characteristics:* importance of knowledge transfer (2 categories), importance of societal engagement (2 categories), press, radio, or television (2 categories), lectures or panel discussions (2 categories), and online social networks (2 categories). “Variables” section provides details. Missing values for the control variables are imputed, and imputation dummies are included in the regressions. The average in the control group is 0.754 for the dummy variable for general support and 0.238 for the dummy variable for strong support

Significance level: *p < 0.1, **p < 0.05, ***p < 0.01

The *Legal duty* treatment has a negative impact on support for an increase in engagement but only with those who strongly support such an increase. With regard to support in general, the treatment has only a negligible and insignificant effect. When focusing on strong support, however, the effect is strongly negative and highly significant. The treatment reduces the probability of being a strong supporter by 6.6 percentage points. With 23.8% of professors in the control group strongly supporting an increase in engagement, this amounts to a drop in the share of strong supporters by 27.7%.

A similar impact derives from the *Expectations public* treatment. With regard to support in general, the treatment has a negative impact that is only marginally significant. The effect on strong support, on the other hand, is highly significant, reducing the probability of being a strong supporter by 6.2 percentage points. This again amounts to a greater than 25% decrease in the share of strong supporters compared to the control group.

There is no discernible impact from the *Support public* treatment. For both support in general and strong support, the treatment effects are small and not significant. In addition, the sign of the treatment indicator’s coefficient is positive in one case and negative in the other.

A negative effect derives from the *Examples risks* treatment. The treatment has a strongly negative and highly significant impact on support for an increase in public engagement in general. It reduces the probability of belonging to the group of supporters by 6.7 percentage points. When compared to the 75.4% of supporters in the control group, this amounts to a decrease in the group of supporters by 8.9%. The treatment’s impact on strong support is smaller and only marginally significant.

### Effect Mechanism

A question remains as to the extent to which the main effects of the treatments derive from the framing or from the information given. This analysis is restricted to the three treatments containing quantifiable information, that is, the *Legal duty* treatment, the *Expectations public* treatment, and the *Support public* treatment. For all three treatments, we elicited the prior knowledge of all respondents before the treatment stage (see Online Appendix Fig. A.1).^6^ These are the estimated number of German federal states stipulating knowledge and technology transfer as a general duty of higher education institutions and the estimated shares of the German population agreeing with the two statements on the relation between science and society from the public opinion poll.^7^

In Table 3, we include the absolute value of the difference between respondents’ elicitation estimate and the true value in the main estimation model. An interaction term between that variable and the treatment indicator should be significantly different from zero if the treatment effect is systematically related to whether the treatment provided respondents with new, relevant knowledge *on average*. As the *Examples risks* treatment is not covered by this analysis, we exclude the observations in this treatment group from the estimation. The results do not show a significant interaction term for any of the combinations of the three treatments and the two outcome variables. Only in the case of the *Support public* treatment in combination with support in general do we observe a significant interaction term, and this interaction term is only marginally significant. Thus, we conclude that the increased salience of the issues emphasized by the treatments is the primary mechanism behind the treatment effects.^8^

**Table 3:** Effects of average respondents’ estimates of treatment information

	General support			Strong support		
	(1)	(2)	(3)	(4)	(5)	(6)
Legal duty	0.004 (0.021)	0.003 (0.021)	0.003 (0.025)	− 0.068*** (0.019)	− 0.064*** (0.019)	− 0.059** (0.024)
Legal duty difference		− 0.001 (0.002)	− 0.001 (0.002)		0.004** (0.002)	0.004** (0.002)
Legal duty × legal duty difference			0.000 (0.004)			− 0.001 (0.004)
Expectations public	− 0.041* (0.021)	− 0.042** (0.021)	− 0.038 (0.029)	− 0.063*** (0.020)	− 0.064*** (0.020)	− 0.092*** (0.026)
Expectations public difference		− 0.001** (0.001)	− 0.001* (0.001)		0.000 (0.001)	0.000 (0.001)
Expectations public × expectations public difference			0.000 (0.001)			0.002 (0.001)
Support public	0.015 (0.020)	0.014 (0.020)	0.044* (0.026)	− 0.026 (0.020)	− 0.027 (0.020)	− 0.025 (0.025)
Support public difference		− 0.001*** (0.000)	− 0.001* (0.001)		0.000 (0.000)	0.000 (0.001)
Support public × support public difference			− 0.002* (0.001)			0.000 (0.001)
Controls	x	x	x	x	x	x
Observations	3,305	3,305	3,305	3,305	3,305	3,305
R-squared	0.064	0.070	0.071	0.065	0.068	0.069

The table shows the coefficients and standard errors of the treatment indicators, variables comprising the absolute value of the difference between respondents’ elicitation estimate of the information provided by the treatments and the true value (columns (2) and (3) and (5) and (6)), and the interaction term between these two variables (columns (3) and (6)) estimated from linear probability models (ordinary least-squares regressions with robust standard errors) regressing the dummy variable indicating respondents’ (strong) support for an increase in engagement on the indicated variables for the subsample consisting of the control group and the treatment groups listed. Full sets of demographic, occupational, and engagement characteristics are always included. See “Variables” section and Online Appendix Table B.1 for details. Missing values for the control variables and elicitation estimates are imputed, and imputation dummies are included in the regressions

Significance level: *p < 0.1, **p < 0.05, ***p < 0.01

### Effect Heterogeneity

In the last step, we analyze treatment effect heterogeneity along individual characteristics (e.g., gender and age), along occupational characteristics (e.g., academic discipline), and along individual attitudes toward science–society relations and the actual engagement in this area (see “Variables” section).^9^ For this, we use the same linear probability models as in the main analysis but estimate them separately for the subgroups. The patterns observed in the subgroup analysis mostly confirm those observed in the main analysis (for an overview of all subgroups, see Online Appendix Tables A.2, A.3 and A.4). Where treatment effects were found in the main analysis, the majority of subgroups show effects in the same direction, even though the effects are not always significant. The same absence of treatment effects found in the main analysis is found for most subgroups as well. However, four differences among the subgroups are pronounced and consistent enough to warrant closer consideration.

**Fig. 3:**
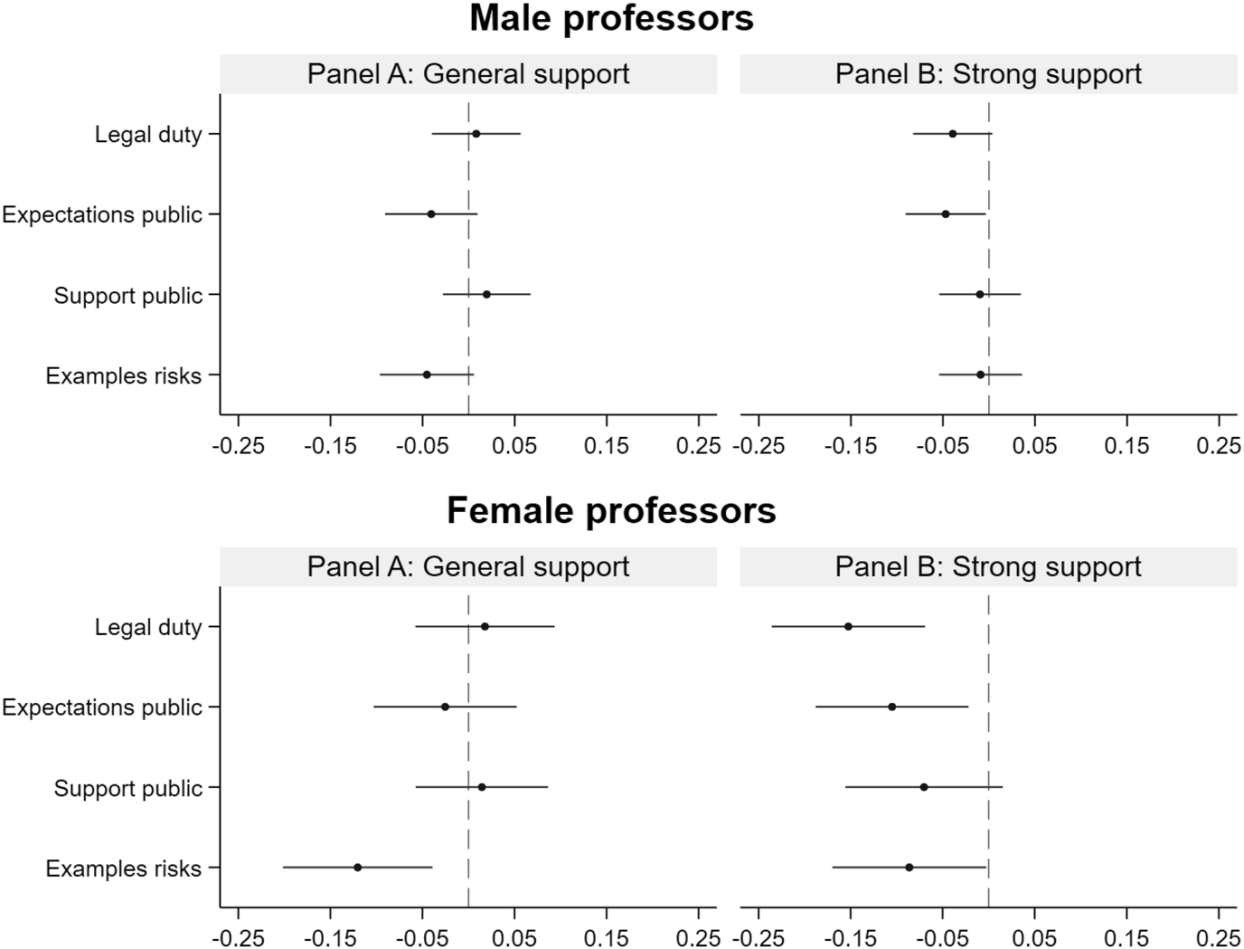
Main effects of the treatments by gender. *Notes* The figure shows gender-specific point estimates and the 95% confidence intervals of the treatment indicators estimated from linear probability models (ordinary least-squares regressions with robust standard errors) regressing the dummy variable indicating respondents’ (strong) support for an increase in engagement on the treatment indicators. Full sets of demographic, occupational, and engagement characteristics are always included. See “Variables” section and Online Appendix Table B.1 for details. Missing values for the control variables (excluding the variable used for the sample split) are imputed, and imputation dummies are included in the regressions. The point estimates represent the change in the probability of (strongly) supporting an increase in academics’ engagement in public discussions due to receipt of the treatment. The average in the control group for the dummy variable for general support is 0.816 for females and 0.732 for males. For the dummy variable for strong support, the control group mean for females is equal to 0.311 and for males 0.207. Number of observations = 983 females and 2985 males. The coefficient plot was produced using the Stata module *coefplot* (Jann, 2014)

A first noteworthy finding is that we observe stronger treatment effects for female professors (see Fig. 3 and Online Appendix Table A.2). Whereas their support for an increase in engagement is higher than that found among male professors, the *Legal duty* treatment and the *Expectations public* treatment both reduce the probability of being strong supporters of an increase in academics’ engagement in public discussions by more than 10 percentage points for female professors. Even if the generally higher levels of support in this group are considered, these effects remain far stronger than the effects for male professors. Set in relation to the share of 31.1% of strong supporters among the female professors in the control group, this amounts to a drop in the share of strong supporters by almost half due to the *Legal duty* treatment and by around one third due to the *Expectations public* treatment. The differences are even more pronounced with regard to the *Examples risks* treatment. For male professors, this treatment has only a marginally significant negative effect on support in general and no effect on strong support. This stands in stark contrast to the strong and significant effects on both general and strong support among female professors. The effects amount to a reduction in the share of supporters in general by 14.7% and in the share of strong supporters by 27.7% if considered in relation to the respective shares among the female professors in the control group.

Online Appendix Table A.2 also shows differences for all three treatments among the different age groups. In the case of the *Legal duty* treatment and the *Expectations public* treatment, the negative effect on strong support is driven by the highest age group of professors (55 years of age or older). This is the only age group for which we observe significant treatment effects in the subgroup analysis, and these effects are also markedly stronger than those in other age groups. The effect of the *Examples risks* treatment is driven by the youngest age group, as it is the only subgroup with a significant effect.^10^ In this group, the treatment reduces the probability of generally supporting an increase in engagement by 15.7 percentage points. Given that 80.8% of those younger than 45 years in the control group support an increase in general, the effect of the *Examples risks* treatment amounts to a drop in the share of supporters in general by 19.4%.

Another pattern emerging from the subgroup analysis is that professors with a positive stance on exchanges with society are particularly strongly affected by two of the three treatments (see Online Appendix Table A.3). Professors who consider knowledge and technology transfer and societal engagement to be very important parts of their job as professors drive the negative impact of the *Legal duty* treatment and the *Expectations public* treatment. The effects of both treatments are highly significant within these two subgroups but are either not significant or only marginally significant for those who consider such activities only somewhat important or not important at all.

In addition, we observe certain differences in treatment effects among the five groups of academic disciplines (see Online Appendix Table A.4). The direction of the treatment effects identified for the pooled sample holds across the groups of disciplines with only one exception. Nevertheless, the effects are driven by three of the five groups: humanities and arts, engineering sciences, and, to a lesser degree, mathematics and natural sciences.^11^ The group of medicine, health sciences and other sciences is rather small, which may partly explain the absence of significant effects. This leaves the comparatively large group of law, economics, social sciences and sport science. The effects in this group constitute the most pronounced deviation from the pattern observed in the main analysis, at least in terms of effect strength. Professors in this group are also those who show the lowest levels of support for an increase in academics’ engagement in public discussions.

## Discussion

Our results show that academics’ attitudes toward engaging in public discussions can indeed be shaped by the prevailing conditions. An emphasis on the indirect demands codified in higher education legislation as well as on expectations voiced by the public induces less-positive attitudes. We also document a negative effect on academics’ attitudes when the risks associated with being exposed to the public are made more salient. In this case, we interpret the result as an upper bound of the effect, as the examples of death threats and lawsuits referred to are extreme examples of the potential negative repercussions. This may also explain why we find an impact of risks, whereas these risks have previously not been found to matter for academics’ engagement with the public (Poliakoff & Webb, 2007). Finally, we do not find an effect from emphasizing high levels of public support for academics’ engagement in public discussions. The absence of an effect of this treatment is underscored by the fact that we do find an effect for the treatment referring to public expectations because these two treatments were designed similarly and reference the same public opinion poll. This raises an interesting question for further research, namely, whether more immediate positive implications of academics’ public engagement, such as potential personal benefits in terms of a greater visibility among peers, are capable of inducing more positive attitudes.

### Opposition to External Demands

A likely explanation for our findings is that external demands and their potential consequences trigger oppositional behavior among professors. Both the demands implicit in higher education legislation and the expectations voiced by the public can be perceived as forces that ultimately shape the immediate working conditions of academics. Given that engaging in public discussions is not necessarily considered a necessary component of academic duties and can even be viewed as being at odds with scientific norms, professors might perceive those demands as an undue infringement on their professional autonomy. Reactions to this in the form of oppositional behavior may be reinforced by the lack of explicit rewards for engaging with the public and insufficient insurance against the risks involved.

Factoring in the differences among the subgroups in terms of professors’ stance on the relations between science and society, it is possible to attribute some oppositional behavior to a crowding out of intrinsic motivation. The negative effects deriving from an emphasis on external demands and expectations concern mainly those who strongly advocate an increase in engagement, and these effects are driven by professors who personally consider it important to engage with those outside of academia. Assuming that these professors exhibit a strong intrinsic motivation to engage, our findings correspond to what is expected from the perspective of motivation crowding theory (Frey, 1997, 2000; Frey & Jegen, 2001). These findings could thus be interpreted as deriving from a conflict between the interventionist character of external demands and professors’ intrinsic motivation to engage with the public. This would explain why we observe strong effects for those who are presumably highly intrinsically motivated but hardly any effects for those who are not. An interpretation from this perspective is particularly reasonable because professors are often assumed to be driven by intrinsic motivation (Osterloh, 2010; Ringelhan et al., 2015). This might be even truer for activities outside the core academic activities (see Dunwoody et al., 2009; Lam, 2015), such as engaging in public discussions.

### Differential Costs Associated with Risks

A closer look at the impact of the risks associated with engaging in public discussions suggests that differences in the associated costs might mediate the strength of such impact. The mere fact that risks, especially the drastic risks referred to by our treatments, have a negative impact on professors’ attitudes is not surprising per se. This makes it even more interesting to observe strong differences depending on the gender and age of professors. With regard to the markedly stronger impact of risks on female professors, it might be conjectured that the hostility they encounter, especially in an online environment, can be comparatively more severe. This is in line with a recent study by Dupas et al. (2021), who find that female presenters in economic conferences and workshops receive more hostile questions than male presenters. It furthermore agrees with findings on differential risk of encountering hostility faced by female and male politicians and the amplifying effect that visibility in the media might have in this regard (Håkansson, 2021). In the case of younger professors, who also react to risks more strongly than their older peers do, the potential costs might also be higher. The negative repercussions of engaging with the public may be perceived as more threatening by younger professors, as they are still building their academic careers and can be affected more strongly by a loss in reputation. Older professors, by contrast, might have already built up a greater reputation as well as a certain amount of resilience, leading them to perceive the risks as less threatening.

### Disciplinary Differences

Differences between groups of academic disciplines furthermore provide indications of an influence of the current state of public engagement in a field, but raise relevant questions for further research as well. By and large, professors from law, economics, social sciences and sport science react less strongly to engagement conditions than professors from other disciplines. They also constitute the group that perceives the least need to increase current levels of engagement. The weaker impact of external demands and risks could, thus, be the result of a greater prevalence and embeddedness of public engagement activities in these disciplines. Nevertheless, whether there are additional reasons for the disciplinary differences observed and why professors from disciplines as different as the humanities and arts and the engineering sciences both react particularly strongly to engagement conditions remain interesting and relevant avenues for further research.

### Generalizability of Findings

Two aspects are worth considering with regard to the broader applicability of our findings. First, it seems that our sample is biased toward professors who are generally more open to engaging with those outside academia. As shown by the subgroup analysis, the professors who are particularly open to such engagement are the most susceptible to the influences we investigate. We thus assume that our sample covers that part of the population of professors that is of interest for the focus of our study, as underrepresented professors would have hardly been affected by the treatments. Second, our investigation was designed as a one-time experiment and we are not able to observe actual changes in the engagement behavior of professors.^12^ However, the conditions covered by our treatments are prevalent in the everyday working life of academics, which makes us confident that the effects we observed are relevant beyond the specific context of our study.

## Conclusion

### Implications for Higher Education Research and Science Studies

The findings of our study contribute to previous research on academics’ engagement with the public and could be relevant for research on relations between science and society more broadly. They show that it is worthwhile to complement research on attitudes as predictors for academics’ engagement with investigations that elucidate how these attitudes are affected. In terms of the conditions of academics’ engagement in public discussions, we identify an initial set of factors that are relevant in this regard. Going beyond this specific area of activities, our findings might also inform studies on academics’ other forms of engagement with the public and the nonacademic environment in general. Several of the activities in this area have recently received greater attention from policy makers and the public, confronting academics with similar demands to those investigated in this study. Given that these activities are not necessarily considered a genuine part of academics’ duties either, the role of intrinsic motivation and related cost considerations are likely to be relevant in this context as well.

Our study also has methodological implications for the field of science and higher education research, as it highlights the usefulness and broader applicability of survey experiments. Adding to those of previous studies, our results confirm that survey experiments are a valuable complement to other methods and extend the scope of issues that can be investigated via survey research. We have furthermore shown that experiments that induce framing effects work with highly educated individuals in scientific fields. This opens up new avenues for investigating attitude formation and decision-making processes in such fields.

### Implications for Designing Incentives and Support Structures

Finally, our results provide several points of reference for science and higher education policy and management. Especially at the policy level, recent attempts to promote academics’ engagement with the nonacademic environment have often been embedded in a specific narrative highlighting the new duties of scientists. Based on our findings, we question whether alluding to such new duties is a sensible strategy, particularly as doing so appears to deter those who are generally open to engagement activities. Such deterrence is relevant with regard to not only the perspective of individual academics but also the broader acceptance of any policy and management initiative in this area.

A more promising strategy to foster relations between the scientific community and the public might include developing institutional incentive and support structures in such a way that they align with rather than counteract the intrinsic motivation of academics. This could comprise measures that reduce the efforts required from academics, for instance, a systematic management of media requests, advice on media interactions and direct support with the development of public statements by public affairs professionals. Presumably even more important would be measures that integrate public engagement activities more closely with the general incentive structures in academia. This could be promoted by considering the time invested in engagement activities as a proper part of academics’ workload and by acknowledging public engagement in hiring and promotion processes. When devising measures, policy makers and managers should take into account that measures addressing all academics in the same way might have different impacts on certain groups of academics, as the gender differences we observe suggest.

### Implications for Managing Risks for Academics

Moreover, universities and public authorities should provide safeguards against the negative repercussions that can derive from academics’ exposure to the public. This seems to be especially warranted for female professors, who seem to be more severely affected by the hostility they may encounter, but also for academics in the early stages of their career. Higher education institutions could prepare academics for challenging situations, for instance, via training programs that explicitly address these situations and how to handle them. Further measures that kick in once such situations arise appear equally important. Possibilities in this regard (see also Nogrady, 2021) include predefined protocols for handling these situations, dedicated contact points within institutions that provide advice and counselling, and technical support with avoiding that offensive and threatening content reaches academics via e-mail or social media. More generally, institutions, policy makers and other stakeholders should recognize the seriousness of this issue and jointly work on strategies to mitigate it. Given that other groups of professors might also be disproportionally deterred by risks, such efforts appear key to promote an adequate representation of the diversity of scientists in the public.

## Supplementary Information

Below is the link to the electronic supplementary material.

Supplementary file1 (DOCX 416 kb)

## Data Availability

The dataset analyzed during the current study is not publicly available for reasons of participant confidentiality, but is available in an anonymized form, together with the code, from the corresponding author on reasonable request.

## Footnotes

1 The experiment is preregistered in the American Economic Association’s registry for randomized controlled trials (AEARCTR-0006565, https://doi.org/10.1257/rct.6565-1.0). IRB approval was received from the Central Ethics Committee of Leibniz University Hannover (EV LUH 18/2020, 17.12.2020).

2 The pretest examined the technical implementation of the survey and the design of the questionnaire. We used insights gained from the pretest to finalize the design of the main item batteries (i.e., to decide upon the number of items and the use of scales versus binary response options), to adapt the response categories and wording of selected questions, and to change the structure of the survey. However, the design of the experiment, including the wording of the questions and treatments as well as the timing of the questions, was not affected by the pretest.

3 In addition to the categories of male and female, respondents could state that they are unable or unwilling to assign themselves to one of these two genders. As only 83 respondents chose this category, which does not allow for a meaningful analysis, such a selection was recoded as a missing value.

4 In the “Effect Heterogeneity” section, we provide some evidence that the treatments are not effective for individuals who have a more critical stance on exchange relations between science and society.

5 As shown in Online Appendix B, the random assignment of respondents to the experimental groups was successful, which allows us to identify the causal effects of the treatments.

6 Due to the structure and requirements of the survey in which the experiment was embedded, the questions eliciting respondents’ prior knowledge were located in the same section as the treatments. Thus, it was possible for respondents to correct their prior estimate after the truth was revealed to them. In Online Appendix E, we discuss this issue and show that the results from this analysis do not qualitatively change when we, for example, exclude observations where the elicitation estimate is exactly the same as the true value.

7 For the analysis, we recoded all impossible values as missing values, that is, estimates of the number of federal states greater than the maximum of 16 (which affected 15 observations) and estimates of the two population shares greater than 100% (which affected 3 and 2 observations, respectively).

8 In further analyses (see Online Appendix D), we estimate the linear probability models of the main analysis separately for subgroups formed by splitting the sample based on the respondents’ elicitation estimates. The results of these analyses do not alter our conclusion that the evidence is too weak to argue that the respondents’ prior level of knowledge is systematically related to the treatment effects.

9 The following subgroup analyses were not prespecified in the preregistration of the experiment.

10 Furthermore, for the youngest age group, we observe a strongly negative and significant effect of the *Expectations public* treatment on support in general, for which we did not observe a treatment effect in the main analysis.

11 For professors in the humanities and arts and in mathematics and natural sciences, we furthermore find a (marginally) significant negative effect of the *Support public* treatment on the strong form of support. We did not observe this effect in the main analysis with the pooled sample.

12 However, we doubt that behavioral changes in the area investigated by us could be observed within a period of time appropriate for a follow-up survey. Moreover, a follow-up survey would likely have suffered from high attrition due to the time constraints and frequent survey requests that professors receive.
