# Supplementary material for: Academics’ Attitudes Toward Engaging in Public Discussions: Experimental Evidence on the Impact of Engagement Conditions
Source: Res High Educ. 2022 Dec 5:1–24. Online ahead of print. doi: 10.1007/s11162-022-09725-4 (PMC9734336; doi:10.1007/s11162-022-09725-4)
Supplement: Supplementary file 1 — Supplementary file1 (DOCX 416 kb) [file 11162_2022_9725_MOESM1_ESM.docx]

# Online Appendix for

*Academics’ Attitudes Toward Engaging in Public Discussions:*

*Experimental Evidence on the Impact of Engagement Conditions*

by Vitus Püttmann^*^, Jens Ruhose, and Stephan L. Thomsen

published in *Research in Higher Education*

**Contents**

A. Figures and Tables 1

B. Balancing Treatment Groups 10

C. Application Survey Weights 14

D. Further Analyses: Effect Mechanism 15

E. Robustness Check: Analysis Effect Mechanism 17

^*^ *Corresponding author*: Vitus Püttmann, Leibniz University Hannover, Institute of Economic Policy, Königsworther Platz 1, 30167 Hannover, Germany, [puettmann@wipol.uni-hannover.de](mailto:puettmann@wipol.uni-hannover.de). ORCID: <https://orcid.org/0000-0002-6152-2764>.

## A. Figures and Tables

**Figure A.1:** Experimental design


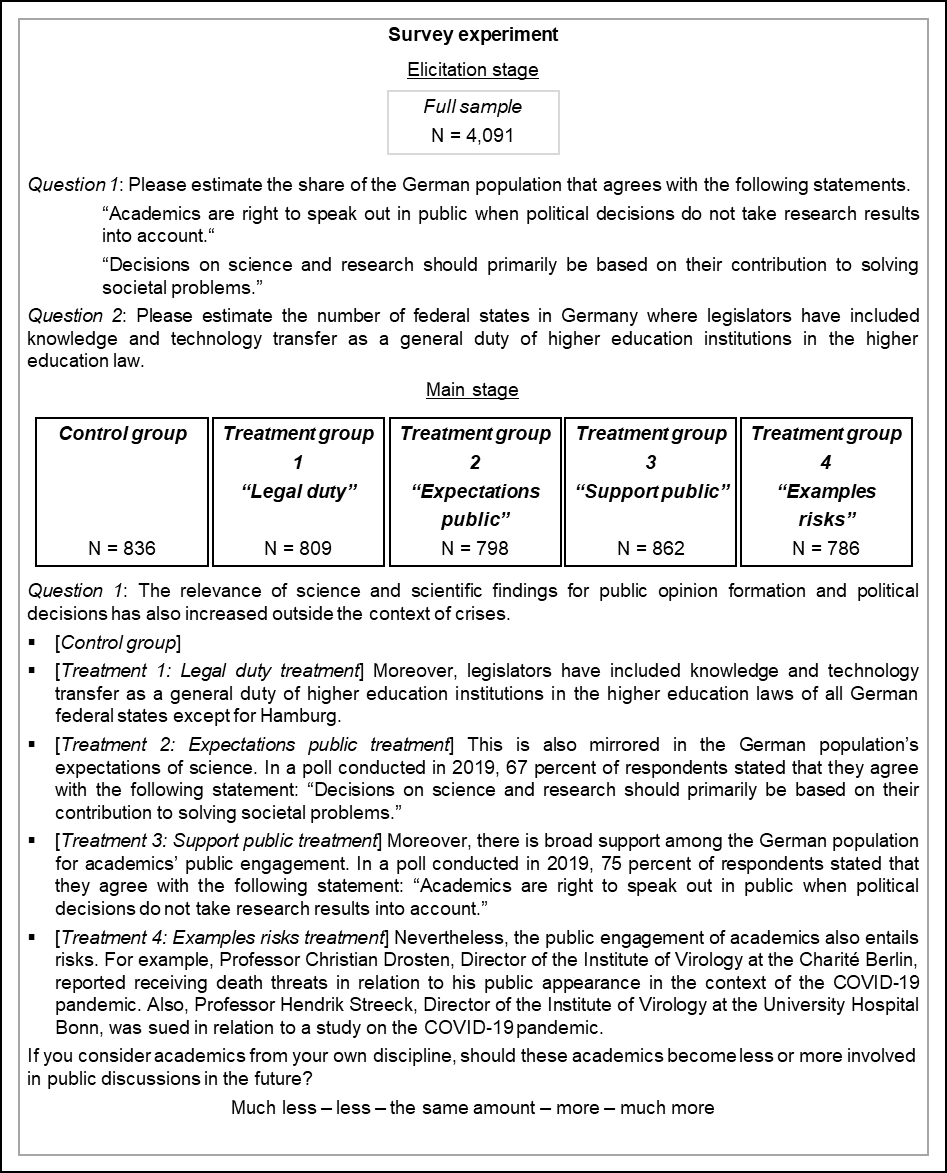


*Notes:* The figure shows the design of the survey experiment. Appendix Figure A.2 shows how the experiment was embedded in the overall survey; Appendix Figure A.3 shows the original German version.

**Figure A.2:** Structure and content of the survey


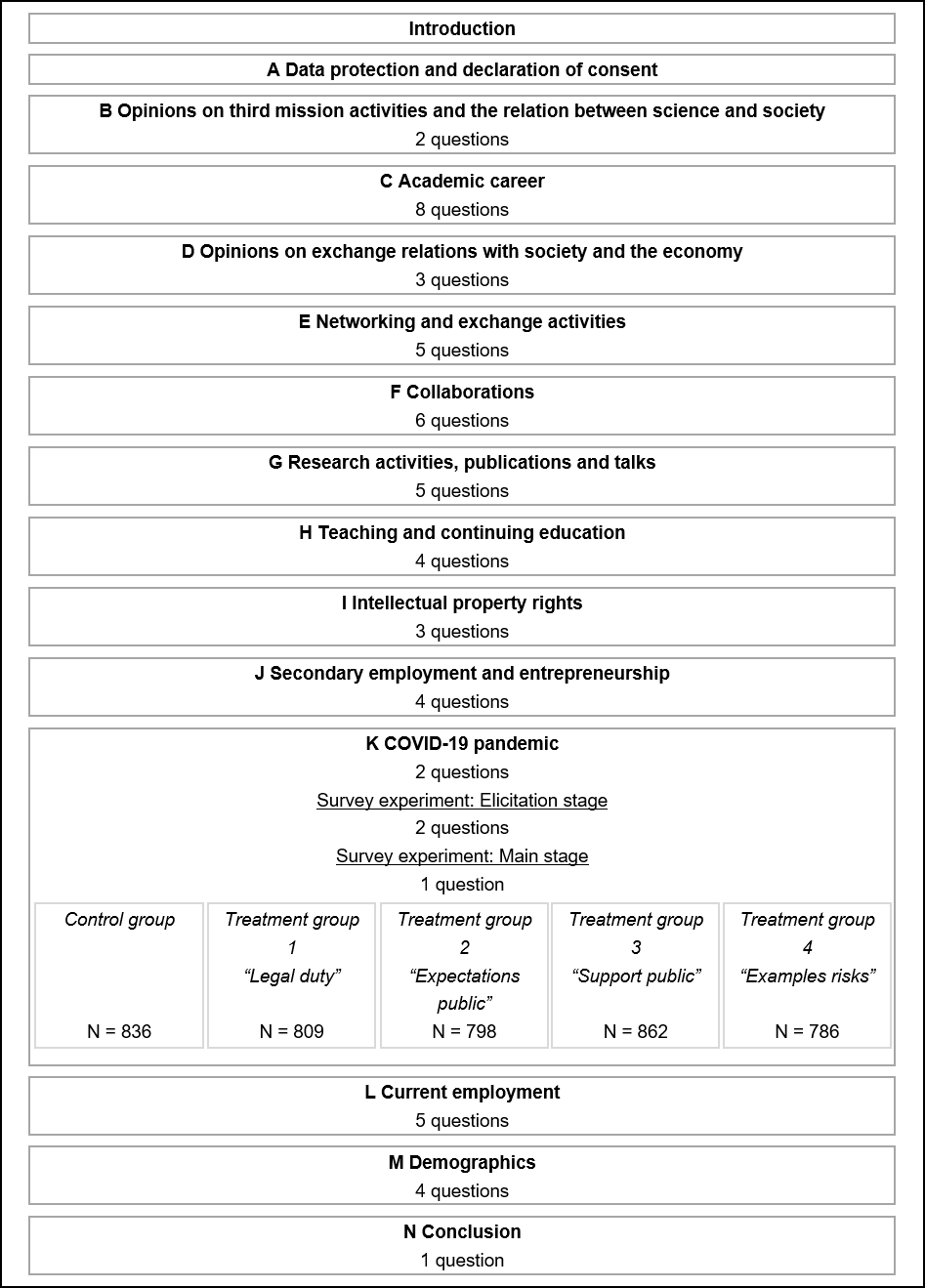


*Notes:* The figure shows the structure and content of the survey, including the number of questions, and details of the experimental design. The number of questions stated includes filtered questions that were shown to a subset of respondents only; some of the questions include multiple items.

**Figure A.3:** Wording of the experiment’s outcome and treatments


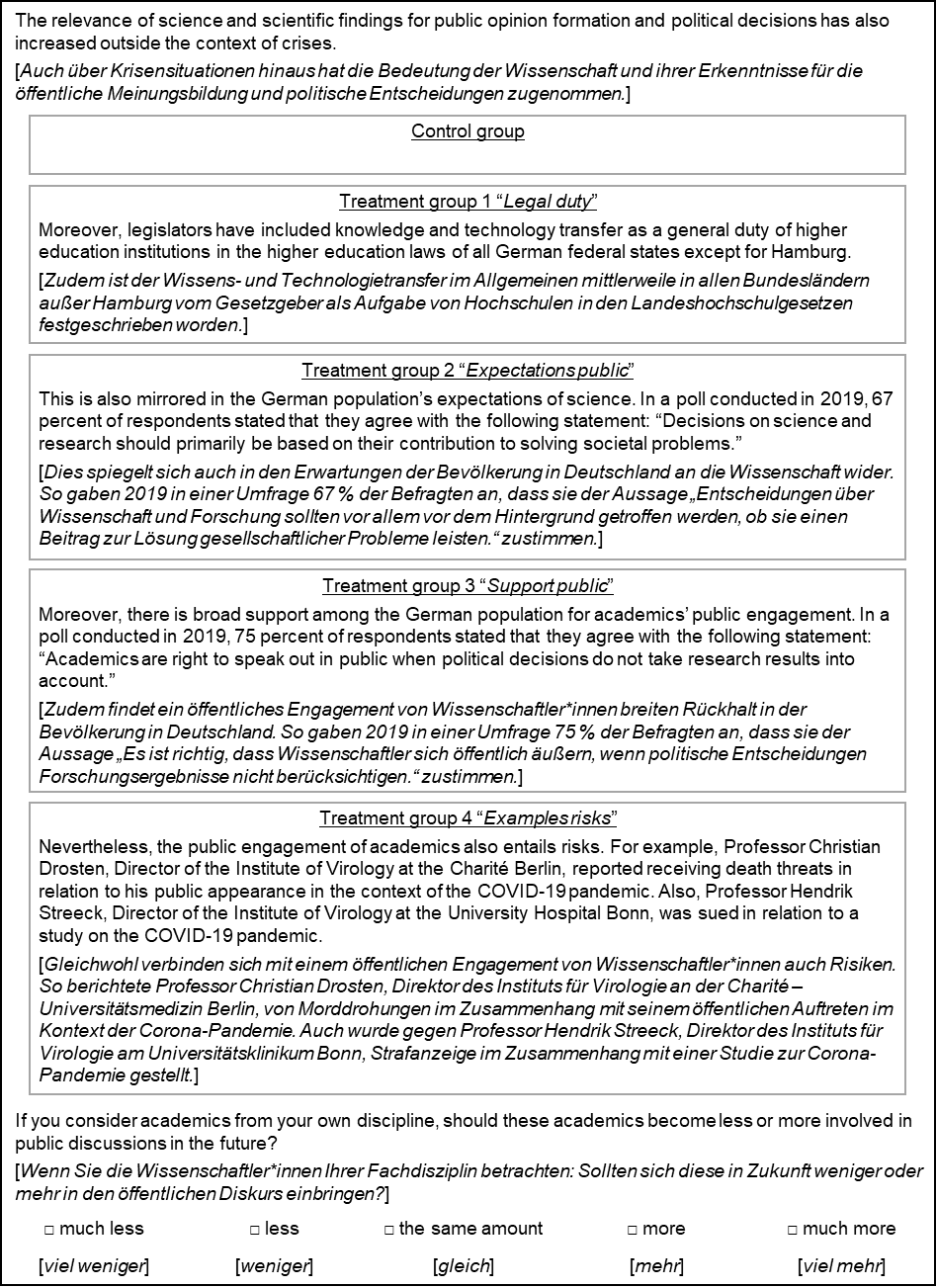


*Notes:* The figure shows the survey question forming the experiment’s outcome and the treatments in English and in the original German version. The treatments included the exact references to the sources of the information provided via a mouseover (see “Experimental Design” section).

**Table A.1:** Comparison of the sample used in the analysis with the APIKS survey sample

| **Sample analysis** | | | | | | |  | **APIKS survey sample** | | | | | |
| --- | --- | --- | --- | --- | --- | --- | --- | --- | --- | --- | --- | --- | --- |
|  | Engineering sciences | Law, economics, social  sciences and sports science | Humanities and arts | Mathematics and  natural sciences | (Veterinary) medicine, health  sciences, agronomy, forestry  and nutrition science | Full sample |  |  | Hard fields of knowledge basic | Hard fields of knowledge applied | Soft fields of knowledge basic | Soft fields of knowledge applied | Full sample |
|  | % | % | % | % | % | % |  |  | % | % | % | % | % |
|  | (1) | (2) | (3) | (4) | (5) | (6) |  |  | (7) | (8) | (9) | (10) | (11) |
| *Year survey* | 2020 | | | | | |  | *Year survey* | 2018 | | | | |
| **Cooperation** | *Involvement previous full year* | | |  |  |  |  | **Cooperation** | *Involvement current / previous year* | | | |  |
| Contract research | 57 | 38 | 17 | 37 | 52 | 41 |  | Contract research | 19 | 41 | 18 | 28 | 28 |
| Consulting | 61 | 63 | 45 | 41 | 65 | 56 |  | Consulting | 26 | 55 | 40 | 52 | 44 |
| **Public engagement** | *Use previous full year* | | |  |  |  |  | **Public engagement** | *Involvement current / previous year* | | | |  |
| Speeches, talks or podium discussions | 66 | 80 | 78 | 58 | 80 | 72 |  | Public speeches or talks | 48 | 55 | 67 | 65 | 59 |
|  |  |  |  |  |  |  |  |  |  |  |  |  |  |
| Books, articles or grey literature | 49 | 72 | 64 | 46 | 72 | 59 |  | Publications for broader audiences | 23 | 26 | 47 | 39 | 34 |
|  |  |  |  |  |  |  |  |  |  |  |  |  |  |

*Notes:* The table shows descriptive statistics of the engagement with the nonacademic environment of professors at public higher education institutions in the sample used in the analysis and in the German survey sample of the research project *Academic Profession in Knowledge Societies* (APIKS) based on Schneijderberg and Götze (2020, 32-33). The table shows the shares of professors engaged in activities in the areas of cooperation and public engagement, both by academic discipline. In the case of the APIKS survey data on third mission activities, scientific disciplines are grouped along two dimensions: the first dimension distinguishes between hard fields of knowledge (i.e., the natural and technical sciences) and soft fields of knowledge (i.e., the humanities and social sciences); the second dimension distinguishes between disciplines oriented toward basic research (e.g, chemistry, German philology, physics and sociology) and disciplines oriented toward application (e.g., business administration, mechanical engineering and social work) (Schneijderberg and Götze 2020, 31). The APIKS survey data includes professors below the associate professorship (i.e., below the W2/C3 salary grade), which are not included in our sample.

**Table A.2:** Effect heterogeneity by demographic characteristics

|  | **Treatment** | | | | | | | | | | | | | | | |  | **Subgroup** | | |
| --- | --- | --- | --- | --- | --- | --- | --- | --- | --- | --- | --- | --- | --- | --- | --- | --- | --- | --- | --- | --- |
|  | *Legal duty* | | | | *Expectations public* | | | | *Support public* | | | | *Examples risks* | | | |  | Control mean | | N |
|  | *General support* | | *Strong support* | | *General support* | | *Strong support* | | *General support* | | *Strong support* | | *General support* | | *Strong support* | |  | *General support* | *Strong support* |  |
|  | (1) | | (2) | | (3) | | (4) | | (5) | | (6) | | (7) | | (8) | |  | (9) | (10) | (11) |
| **Gender** |  |  |  |  |  |  |  |  |  |  |  |  |  |  |  |  |  |  |  |  |
| *Male* | 0.008 |  | -0.039 | * | -0.041 |  | -0.047 | ** | 0.020 |  | -0.010 |  | -0.045 | * | -0.009 |  |  | 0.732 | 0.207 | 2,985 |
|  | (0.025) |  | (0.022) |  | (0.026) |  | (0.022) |  | (0.024) |  | (0.023) |  | (0.026) |  | (0.023) |  |  |  |  |  |
| *Female* | 0.018 |  | -0.152 | *** | -0.025 |  | -0.105 | ** | 0.015 |  | -0.070 |  | -0.120 | *** | -0.086 | ** |  | 0.816 | 0.311 | 983 |
|  | (0.039) |  | (0.042) |  | (0.040) |  | (0.042) |  | (0.037) |  | (0.044) |  | (0.041) |  | (0.043) |  |  |  |  |  |
| **Age group** |  |  |  |  |  |  |  |  |  |  |  |  |  |  |  |  |  |  |  |  |
| *< 45 years* | -0.056 |  | -0.027 |  | -0.125 | ** | -0.032 |  | -0.060 |  | -0.057 |  | -0.157 | *** | -0.014 |  |  | 0.808 | 0.217 | 603 |
|  | (0.054) |  | (0.053) |  | (0.058) |  | (0.052) |  | (0.052) |  | (0.050) |  | (0.059) |  | (0.053) |  |  |  |  |  |
| *45-54 years* | 0.039 |  | -0.042 |  | -0.026 |  | -0.050 |  | 0.039 |  | 0.019 |  | -0.053 |  | -0.025 |  |  | 0.742 | 0.227 | 1,565 |
|  | (0.034) |  | (0.031) |  | (0.035) |  | (0.031) |  | (0.032) |  | (0.032) |  | (0.035) |  | (0.032) |  |  |  |  |  |
| *> 54 years* | 0.003 |  | -0.086 | *** | -0.029 |  | -0.076 | ** | 0.030 |  | -0.050 |  | -0.053 |  | -0.041 |  |  | 0.755 | 0.255 | 1,833 |
|  | (0.031) |  | (0.029) |  | (0.032) |  | (0.030) |  | (0.030) |  | (0.030) |  | (0.032) |  | (0.031) |  |  |  |  |  |

*Notes:* In columns (1) to (8), the table shows the coefficients and standard errors of the treatment indicators estimated from linear probability models (ordinary least-squares regressions with robust standard errors) regressing the dummy variable indicating respondents’ (strong) support for an increase in engagement on the treatment indicators. Full sets of demographic, occupational, and engagement characteristics are always included. See “Variables” section and Appendix Table B.1 for details. Missing values for the control variables (excluding those used for the sample split) are imputed, and imputation dummies are included in the regressions. In columns (9) to (11), the table shows the mean of the dummy variable indicating respondents’ (strong) support and the number of observations in the subgroup. Significance level: * p < 0.1, ** p < 0.05, *** p < 0.01.

**Table A.3:** Effect heterogeneity by engagement characteristics

|  | **Treatment** | | | | | | | | | | | | | | | |  | **Subgroup** | | |
| --- | --- | --- | --- | --- | --- | --- | --- | --- | --- | --- | --- | --- | --- | --- | --- | --- | --- | --- | --- | --- |
|  | *Legal duty* | | | | *Expectations public* | | | | *Support public* | | | | *Examples risks* | | | |  | Control mean | | N |
|  | *General support* | | *Strong support* | | *General support* | | *Strong support* | | *General support* | | *Strong support* | | *General support* | | *Strong support* | |  | *General support* | *Strong support* |  |
|  | (1) | | (2) | | (3) | | (4) | | (5) | | (6) | | (7) | | (8) | |  | (9) | (10) | (11) |
| **Importance knowledge transfer** | | | | | | | | | | | | | | | | |  |  |  |  |
| *Not / somewhat important* | 0.004 |  | -0.042 |  | -0.036 |  | -0.056 | * | 0.036 |  | 0.008 |  | -0.074 |  | -0.036 |  |  | 0.650 | 0.142 | 1,098 |
|  | (0.044) |  | (0.030) |  | (0.046) |  | (0.030) |  | (0.044) |  | (0.034) |  | (0.046) |  | (0.031) |  |  |  |  |  |
| *Very important* | 0.002 |  | -0.080 | *** | -0.044 | * | -0.071 | *** | 0.006 |  | -0.038 |  | -0.070 | *** | -0.036 |  |  | 0.794 | 0.275 | 2,971 |
|  | (0.023) |  | (0.024) |  | (0.024) |  | (0.024) |  | (0.022) |  | (0.024) |  | (0.025) |  | (0.025) |  |  |  |  |  |
| **Importance societal engagement** | | | | | | | | | | | | | | | | |  |  |  |  |
| *Not / somewhat important* | 0.008 |  | -0.042 | * | -0.032 |  | -0.038 |  | -0.004 |  | -0.011 |  | -0.089 | ** | -0.022 |  |  | 0.675 | 0.138 | 1,835 |
|  | (0.034) |  | (0.024) |  | (0.034) |  | (0.024) |  | (0.033) |  | (0.025) |  | (0.035) |  | (0.024) |  |  |  |  |  |
| *Very important* | -0.001 |  | -0.083 | *** | -0.049 | * | -0.077 | *** | 0.023 |  | -0.034 |  | -0.053 | * | -0.046 |  |  | 0.822 | 0.322 | 2,236 |
|  | (0.025) |  | (0.029) |  | (0.027) |  | (0.030) |  | (0.024) |  | (0.030) |  | (0.027) |  | (0.031) |  |  |  |  |  |
| **Press, radio or television** | | | | | | | | | | | | | | | | |  |  |  |  |
| *No* | -0.016 |  | -0.078 | *** | -0.046 |  | -0.055 | ** | 0.010 |  | -0.042 |  | -0.067 | ** | -0.019 |  |  | 0.736 | 0.205 | 2,086 |
|  | (0.030) |  | (0.025) |  | (0.030) |  | (0.025) |  | (0.029) |  | (0.026) |  | (0.031) |  | (0.027) |  |  |  |  |  |
| *Yes* | 0.019 |  | -0.064 | ** | -0.033 |  | -0.083 | *** | 0.024 |  | -0.026 |  | -0.069 | ** | -0.053 | * |  | 0.781 | 0.285 | 1,932 |
|  | (0.028) |  | (0.031) |  | (0.030) |  | (0.030) |  | (0.028) |  | (0.031) |  | (0.031) |  | (0.031) |  |  |  |  |  |
| **Lectures or panel discussions** | | | | | | | | | | | | | | | | |  |  |  |  |
| *No* | -0.011 |  | -0.079 | ** | -0.025 |  | -0.088 | *** | 0.037 |  | -0.044 |  | -0.030 |  | -0.017 |  |  | 0.669 | 0.189 | 1,132 |
|  | (0.042) |  | (0.031) |  | (0.044) |  | (0.031) |  | (0.042) |  | (0.034) |  | (0.043) |  | (0.034) |  |  |  |  |  |
| *Yes* | 0.009 |  | -0.060 | ** | -0.047 | * | -0.051 | ** | 0.012 |  | -0.021 |  | -0.076 | *** | -0.039 |  |  | 0.790 | 0.260 | 2,918 |
|  | (0.023) |  | (0.024) |  | (0.024) |  | (0.024) |  | (0.023) |  | (0.024) |  | (0.025) |  | (0.025) |  |  |  |  |  |

*(continued on next page)*

**Table A.3 (continued)**

|  | **Treatment** | | | | | | | | | | | | | | | |  | **Subgroup** | | |
| --- | --- | --- | --- | --- | --- | --- | --- | --- | --- | --- | --- | --- | --- | --- | --- | --- | --- | --- | --- | --- |
|  | *Legal duty* | | | | *Expectations public* | | | | *Support public* | | | | *Examples risks* | | | |  | Control mean | | N |
|  | *General support* | | *Strong support* | | *General support* | | *Strong support* | | *General support* | | *Strong support* | | *General support* | | *Strong support* | |  | *General support* | *Strong support* |  |
|  | (1) | | (2) | | (3) | | (4) | | (5) | | (6) | | (7) | | (8) | |  | (9) | (10) | (11) |
| **Online social networks** | | | | | | | | | | | | | | | | |  |  |  |  |
| *No* | -0.007 |  | -0.072 | *** | -0.025 |  | -0.075 | *** | 0.011 |  | -0.048 | ** | -0.060 | ** | -0.040 | * |  | 0.738 | 0.235 | 2,880 |
|  | (0.025) |  | (0.023) |  | (0.025) |  | (0.023) |  | (0.025) |  | (0.023) |  | (0.026) |  | (0.024) |  |  |  |  |  |
| *Yes* | 0.007 |  | -0.071 | * | -0.095 | ** | -0.040 |  | 0.020 |  | -0.001 |  | -0.080 | ** | -0.024 |  |  | 0.811 | 0.249 | 1,108 |
|  | (0.036) |  | (0.038) |  | (0.041) |  | (0.041) |  | (0.035) |  | (0.040) |  | (0.040) |  | (0.041) |  |  |  |  |  |

*Notes:* In columns (1) to (8), the table shows the coefficients and standard errors of the treatment indicators estimated from linear probability models (ordinary least-squares regressions with robust standard errors) regressing the dummy variable indicating respondents’ (strong) support for an increase in engagement on the treatment indicators. Full sets of demographic, occupational, and engagement characteristics are always included. See “Variables” section and Appendix Table B.1 for details. Missing values for the control variables (excluding those used for the sample split) are imputed, and imputation dummies are included in the regressions. In columns (9) to (11), the table shows the mean of the dummy variable indicating respondents’ (strong) support and the number of observations in the subgroup. Significance level: * p < 0.1, ** p < 0.05, *** p < 0.01.

**Table A.4:** Effect heterogeneity by occupational characteristics

|  | **Treatment** | | | | | | | | | | | | | | | |  | **Subgroup** | | |
| --- | --- | --- | --- | --- | --- | --- | --- | --- | --- | --- | --- | --- | --- | --- | --- | --- | --- | --- | --- | --- |
|  | *Legal duty* | | | | *Expectations public* | | | | *Support public* | | | | *Examples risks* | | | |  | Control mean | | N |
|  | *General support* | | *Strong support* | | *General support* | | *Strong support* | | *General support* | | *Strong support* | | *General support* | | *Strong support* | |  | *General support* | *Strong support* |  |
|  | (1) | | (2) | | (3) | | (4) | | (5) | | (6) | | (7) | | (8) | |  | (9) | (10) | (11) |
| **Type of professorship** | | | | | | | | | | | | | | | | |  |  |  |  |
| *Associate professor* | 0.005 |  | -0.071 | *** | -0.037 |  | -0.060 | ** | 0.008 |  | -0.030 |  | -0.059 | ** | -0.024 |  |  | 0.762 | 0.249 | 2,626 |
|  | (0.025) |  | (0.024) |  | (0.026) |  | (0.025) |  | (0.025) |  | (0.025) |  | (0.026) |  | (0.025) |  |  |  |  |  |
| *Full professor* | 0.010 |  | -0.055 | * | -0.036 |  | -0.062 | ** | 0.034 |  | -0.015 |  | -0.083 | ** | -0.055 | * |  | 0.737 | 0.218 | 1,465 |
|  | (0.036) |  | (0.032) |  | (0.037) |  | (0.032) |  | (0.034) |  | (0.032) |  | (0.039) |  | (0.033) |  |  |  |  |  |
| **Group of academic disciplines** | | | | | | | | | | | | | | | | |  |  |  |  |
| *Humanities and arts* | -0.025 |  | -0.147 | *** | 0.002 |  | -0.088 | * | -0.026 |  | -0.097 | ** | -0.107 | * | -0.093 | * |  | 0.776 | 0.297 | 696 |
|  | (0.050) |  | (0.047) |  | (0.048) |  | (0.048) |  | (0.047) |  | (0.049) |  | (0.055) |  | (0.051) |  |  |  |  |  |
| *Law, economics, social sc.* | 0.056 |  | -0.044 |  | -0.026 |  | -0.017 |  | 0.047 |  | 0.057 |  | -0.027 |  | 0.046 |  |  | 0.698 | 0.187 | 1,160 |
|  | (0.042) |  | (0.035) |  | (0.042) |  | (0.035) |  | (0.040) |  | (0.037) |  | (0.042) |  | (0.037) |  |  |  |  |  |
| *Mathematics and natural sc.* | -0.069 |  | -0.057 |  | -0.108 | * | -0.101 | ** | -0.016 |  | -0.080 | * | -0.050 |  | -0.026 |  |  | 0.779 | 0.229 | 635 |
|  | (0.050) |  | (0.049) |  | (0.059) |  | (0.049) |  | (0.050) |  | (0.048) |  | (0.050) |  | (0.049) |  |  |  |  |  |
| *Medicine, health sc. and others* | 0.029 |  | 0.015 |  | 0.068 |  | -0.038 |  | 0.050 |  | -0.030 |  | -0.075 |  | 0.045 |  |  | 0.746 | 0.237 | 334 |
|  | (0.078) |  | (0.079) |  | (0.076) |  | (0.077) |  | (0.068) |  | (0.074) |  | (0.083) |  | (0.083) |  |  |  |  |  |
| *Engineering sciences* | 0.008 |  | -0.084 | ** | -0.059 |  | -0.092 | ** | 0.016 |  | -0.029 |  | -0.084 | ** | -0.101 | *** |  | 0.782 | 0.250 | 1,243 |
|  | (0.036) |  | (0.035) |  | (0.038) |  | (0.036) |  | (0.037) |  | (0.038) |  | (0.040) |  | (0.036) |  |  |  |  |  |

*(continued on next page)*

**Table A.4 (continued)**

|  | **Treatment** | | | | | | | | | | | | | | | |  | **Subgroup** | | |
| --- | --- | --- | --- | --- | --- | --- | --- | --- | --- | --- | --- | --- | --- | --- | --- | --- | --- | --- | --- | --- |
|  | *Legal duty* | | | | *Expectations public* | | | | *Support public* | | | | *Examples risks* | | | |  | Control mean | | N |
|  | *General support* | | *Strong support* | | *General support* | | *Strong support* | | *General support* | | *Strong support* | | *General support* | | *Strong support* | |  | *General support* | *Strong support* |  |
|  | (1) | | (2) | | (3) | | (4) | | (5) | | (6) | | (7) | | (8) | |  | (9) | (10) | (11) |
| **Type of institution** | | | | | | | | | | | | | | | | |  |  |  |  |
| *University* | -0.001 |  | -0.060 | ** | -0.054 | * | -0.076 | *** | 0.028 |  | -0.026 |  | -0.080 | ** | -0.030 |  |  | 0.729 | 0.206 | 1,841 |
|  | (0.032) |  | (0.028) |  | (0.033) |  | (0.027) |  | (0.030) |  | (0.028) |  | (0.033) |  | (0.029) |  |  |  |  |  |
| *University of applied sciences* | 0.016 |  | -0.079 | *** | -0.025 |  | -0.060 | ** | 0.007 |  | -0.033 |  | -0.073 | ** | -0.053 | * |  | 0.778 | 0.271 | 1,930 |
|  | (0.029) |  | (0.029) |  | (0.030) |  | (0.030) |  | (0.029) |  | (0.031) |  | (0.031) |  | (0.030) |  |  |  |  |  |
| *College of art / music* | 0.096 |  | -0.111 |  | 0.067 |  | -0.022 |  | -0.030 |  | -0.123 |  | -0.077 |  | -0.134 |  |  | 0.787 | 0.319 | 207 |
|  | (0.083) |  | (0.098) |  | (0.090) |  | (0.106) |  | (0.091) |  | (0.100) |  | (0.101) |  | (0.097) |  |  |  |  |  |
| **Region** | | | | | | | | | | | | | | | | |  |  |  |  |
| *West Germany* | 0.014 |  | -0.048 | ** | -0.038 |  | -0.064 | *** | 0.030 |  | -0.025 |  | -0.064 | *** | -0.037 | * |  | 0.748 | 0.230 | 3,156 |
|  | (0.023) |  | (0.022) |  | (0.024) |  | (0.022) |  | (0.023) |  | (0.023) |  | (0.025) |  | (0.023) |  |  |  |  |  |
| *East Germany* | -0.011 |  | -0.133 | *** | -0.047 |  | -0.044 |  | -0.044 |  | -0.041 |  | -0.103 | ** | -0.024 |  |  | 0.786 | 0.272 | 821 |
|  | (0.046) |  | (0.042) |  | (0.046) |  | (0.046) |  | (0.046) |  | (0.045) |  | (0.050) |  | (0.048) |  |  |  |  |  |

*Notes:* In columns (1) to (8), the table shows the coefficients and standard errors of the treatment indicators estimated from linear probability models (ordinary least-squares regressions with robust standard errors) regressing the dummy variable indicating respondents’ (strong) support for an increase in engagement on the treatment indicators. Full sets of demographic, occupational, and engagement characteristics are always included. See “Variables” section and Appendix Table B.1 for details. Missing values for the control variables (excluding those used for the sample split) are imputed, and imputation dummies are included in the regressions. In columns (9) to (11), the table shows the mean of the dummy variable indicating respondents’ (strong) support and the number of observations in the subgroup. “Law, economics, social sc.” includes sport science; “sc.” = science(s). Significance level: * p < 0.1, ** p < 0.05, *** p < 0.01.

## B. Balancing Treatment Groups

Two balance checks confirm that the random assignment of respondents to the experimental groups was successful. In the first check, we test whether the composition of the treatment groups in terms of observable characteristics differs from that of the control group. We do this by regressing each of the covariates in our set of control variables separately on each of the four treatment indicators, using the subsamples consisting of the control group and the respective treatment group. Out of the 108 coefficients in the estimations (see Appendix Table B.1), 5.6 percent are significant at a 5 percent confidence level, which is similar to what would be expected by chance. In the second check, we regress each of the four treatment indicators on all control variables together, again using the subsamples consisting of the control group and the respective treatment group. In all four cases, the F-test for joint significance of all covariates is insignificant, and the coefficients of determination are close to zero (see Appendix Table B.1). We can thus infer that there is no systematic variation in the observable and unobservable characteristics among the experimental groups, which allows us to identify the causal effects of the treatments.

**Table B.1:** Descriptive statistics and covariate balancing

|  | **Control mean** | **Treatment** | | | | | | | |
| --- | --- | --- | --- | --- | --- | --- | --- | --- | --- |
|  |  | *Legal*  *duty* | | *Expectations*  *public* | | *Support*  *public* | | *Examples*  *risks* | |
|  | (1) | (2) | | (3) | | (4) | | (5) | |
| **Preferred extent of engagement in public discussions (outcome)** | | | | | | | | | |
| *Much less* | 0.007 | -0.002 |  | -0.001 |  | 0.001 |  | 0.002 |  |
|  |  | (0.004) |  | (0.004) |  | (0.004) |  | (0.004) |  |
| *Less* | 0.024 | -0.004 |  | 0.004 |  | -0.004 |  | 0.003 |  |
|  |  | (0.007) |  | (0.008) |  | (0.007) |  | (0.008) |  |
| *The same amount* | 0.215 | 0.000 |  | 0.029 |  | -0.020 |  | 0.063 | ***** |
|  |  | (0.020) |  | (0.021) |  | (0.020) |  | (0.021) |  |
| *More* | 0.516 | 0.072 | ***** | 0.026 |  | 0.044 | *** | -0.028 |  |
|  |  | (0.024) |  | (0.025) |  | (0.024) |  | (0.025) |  |
| *Much more* | 0.238 | -0.065 | ***** | -0.058 | ***** | -0.020 |  | -0.040 | *** |
|  |  | (0.020) |  | (0.020) |  | (0.020) |  | (0.020) |  |
| Observations | 836 | 809 |  | 798 |  | 862 |  | 786 |  |
| **Gender** | | | | | | | | | |
| *Male* | 0.738 | 0.049 | **** | 0.015 |  | 0.004 |  | 0.003 |  |
|  |  | (0.021) |  | (0.022) |  | (0.022) |  | (0.022) |  |
| *Female* | 0.262 | -0.049 | ** | -0.015 |  | -0.004 |  | -0.003 |  |
|  |  | (0.021) |  | (0.022) |  | (0.022) |  | (0.022) |  |
| Observations | 810 | 780 |  | 783 |  | 830 |  | 765 |  |
| **Age group** | | | | | | | | | |
| *< 45 years* | 0.147 | 0.001 |  | 0.001 |  | 0.009 |  | 0.004 |  |
|  |  | (0.018) |  | (0.018) |  | (0.018) |  | (0.018) |  |
| *45-54 years* | 0.401 | -0.017 |  | -0.012 |  | -0.004 |  | -0.014 |  |
|  |  | (0.024) |  | (0.024) |  | (0.024) |  | (0.025) |  |
| *> 54 years* | 0.452 | 0.016 |  | 0.011 |  | -0.006 |  | 0.010 |  |
|  |  | (0.025) |  | (0.025) |  | (0.024) |  | (0.025) |  |
| Observations | 814 | 793 |  | 786 |  | 842 |  | 766 |  |
| **Type of professorship** | | | | | | | | | |
| *Associate professor* | 0.659 | -0.006 |  | -0.036 |  | -0.041 | * | -0.001 |  |
|  |  | (0.023) |  | (0.024) |  | (0.023) |  | (0.024) |  |
| *Full professor* | 0.341 | 0.006 |  | 0.036 |  | 0.041 | * | 0.001 |  |
|  |  | (0.023) |  | (0.024) |  | (0.023) |  | (0.024) |  |
| Observations | 836 | 809 |  | 798 |  | 862 |  | 786 |  |

*(continued on next page)*

**Table B.1 (continued)**

|  | **Control mean** | **Treatment** | | | | | | | |
| --- | --- | --- | --- | --- | --- | --- | --- | --- | --- |
|  |  | *Legal*  *duty* | | *Expectations*  *public* | | *Support*  *public* | | *Examples*  *risks* | |
|  | (1) | (2) | | (3) | | (4) | | (5) | |
| **Group of academic disciplines** | | | | | | | | | |
| *Engineering sciences* | 0.303 | 0.026 |  | 0.010 |  | -0.025 |  | 0.004 |  |
|  |  | (0.023) |  | (0.023) |  | (0.022) |  | (0.023) |  |
| *Law, economics, social sciences and sports science* | 0.270 | -0.005 |  | 0.035 |  | 0.020 |  | 0.024 |  |
|  |  | (0.022) |  | (0.022) |  | (0.022) |  | (0.022) |  |
| *Humanities and arts* | 0.198 | -0.035 | * | -0.016 |  | -0.030 |  | -0.056 | *** |
|  |  | (0.019) |  | (0.019) |  | (0.019) |  | (0.019) |  |
| *Mathematics and natural sciences* | 0.158 | -0.003 |  | -0.033 | * | 0.005 |  | 0.024 |  |
|  |  | (0.018) |  | (0.017) |  | (0.018) |  | (0.019) |  |
| *Medicine, health sciences and  others* | 0.071 | 0.018 |  | 0.003 |  | 0.031 | ** | 0.003 |  |
|  |  | (0.013) |  | (0.013) |  | (0.014) |  | (0.013) |  |
| Observations | 832 | 803 |  | 795 |  | 857 |  | 781 |  |
| **Type of institution** |  |  |  |  |  |  |  |  |  |
| *University* | 0.457 | -0.001 |  | 0.004 |  | 0.027 |  | -0.004 |  |
|  |  | (0.025) |  | (0.025) |  | (0.025) |  | (0.025) |  |
| *University of applied sciences* | 0.485 | 0.009 |  | 0.012 |  | -0.021 |  | 0.006 |  |
|  |  | (0.025) |  | (0.025) |  | (0.025) |  | (0.025) |  |
| *College of art / music* | 0.058 | -0.008 |  | -0.016 |  | -0.006 |  | -0.002 |  |
|  |  | (0.011) |  | (0.011) |  | (0.011) |  | (0.012) |  |
| Observations | 807 | 791 |  | 784 |  | 835 |  | 761 |  |
| **Region** |  |  |  |  |  |  |  |  |  |
| *West Germany* | 0.786 | 0.016 |  | 0.004 |  | 0.003 |  | 0.015 |  |
|  |  | (0.020) |  | (0.021) |  | (0.020) |  | (0.020) |  |
| *East Germany* | 0.214 | -0.016 |  | -0.004 |  | -0.003 |  | -0.015 |  |
|  |  | (0.020) |  | (0.021) |  | (0.020) |  | (0.020) |  |
| Observations | 809 | 783 |  | 781 |  | 838 |  | 766 |  |
| **Importance knowledge transfer** |  |  |  |  |  |  |  |  |  |
| *Not / somewhat important* | 0.271 | 0.009 |  | -0.012 |  | -0.014 |  | 0.012 |  |
|  |  | (0.022) |  | (0.022) |  | (0.021) |  | (0.022) |  |
| *Very important* | 0.729 | -0.009 |  | 0.012 |  | 0.014 |  | -0.012 |  |
|  |  | (0.022) |  | (0.022) |  | (0.021) |  | (0.022) |  |
| Observations | 833 | 804 |  | 792 |  | 859 |  | 781 |  |
| **Importance societal engagement** |  |  |  |  |  |  |  |  |  |
| *Not / somewhat important* | 0.460 | -0.019 |  | -0.020 |  | -0.022 |  | 0.013 |  |
|  |  | (0.025) |  | (0.025) |  | (0.024) |  | (0.025) |  |
| *Very important* | 0.540 | 0.019 |  | 0.020 |  | 0.022 |  | -0.013 |  |
|  |  | (0.025) |  | (0.025) |  | (0.024) |  | (0.025) |  |
| Observations | 834 | 804 |  | 791 |  | 859 |  | 783 |  |

*(continued on next page)*

**Table B.1 (continued)**

|  | **Control mean** | **Treatment** | | | | | | | |
| --- | --- | --- | --- | --- | --- | --- | --- | --- | --- |
|  |  | *Legal*  *duty* | | *Expectations*  *public* | | *Support*  *public* | | *Examples*  *risks* | |
|  | (1) | (2) | | (3) | | (4) | | (5) | |
| **Press, radio or television** |  |  |  |  |  |  |  |  |  |
| *No* | 0.535 | -0.017 |  | -0.029 |  | -0.032 |  | 0.002 |  |
|  |  | (0.025) |  | (0.025) |  | (0.024) |  | (0.025) |  |
| *Yes* | 0.465 | 0.017 |  | 0.029 |  | 0.032 |  | -0.002 |  |
|  |  | (0.025) |  | (0.025) |  | (0.024) |  | (0.025) |  |
| Observations | 823 | 792 |  | 789 |  | 846 |  | 768 |  |
| **Lectures or panel discussions** |  |  |  |  |  |  |  |  |  |
| *No* | 0.306 | -0.018 |  | -0.042 | * | -0.054 | ** | -0.018 |  |
|  |  | (0.023) |  | (0.022) |  | (0.022) |  | (0.023) |  |
| *Yes* | 0.694 | 0.018 |  | 0.042 | * | 0.054 | ** | 0.018 |  |
|  |  | (0.023) |  | (0.022) |  | (0.022) |  | (0.023) |  |
| Observations | 830 | 801 |  | 791 |  | 853 |  | 775 |  |
| **Online social networks** |  |  |  |  |  |  |  |  |  |
| *No* | 0.733 | -0.023 |  | 0.001 |  | -0.028 |  | -0.002 |  |
|  |  | (0.022) |  | (0.022) |  | (0.022) |  | (0.022) |  |
| *Yes* | 0.267 | 0.023 |  | -0.001 |  | 0.028 |  | 0.002 |  |
|  |  | (0.022) |  | (0.022) |  | (0.022) |  | (0.022) |  |
| Observations | 812 | 790 |  | 778 |  | 843 |  | 765 |  |
| **F-test joint significance** |  |  |  |  |  |  |  |  |  |
| p-value |  | 0.830 |  | 0.485 |  | 0.267 |  | 0.133 |  |
| Observations |  | 1,449 |  | 1,456 |  | 1,499 |  | 1,427 |  |
| Adj. R-squared |  | -0.004 |  | 0.000 |  | 0.002 |  | 0.004 |  |

*Notes:* The table shows the coefficients and robust standard errors from separate ordinary least-squares regressions of the covariates listed on the treatment indicators for the subsamples consisting of the control group and the respective treatment group, and the number of observations in the respective treatment group included in these estimations in columns (2) to (5), and the means of the variables and the number of observations for the control group in column (1). The coefficients represent the differences in the mean between the control group and the respective treatment group. At the bottom, the table shows summary statistics for F-tests on the joint significance of the covariates when regressing the treatment indicator on all control variables for the subsample consisting of the control group and the respective treatment group. “others” includes agronomy, forestry, nutrition science and veterinary medicine. Significance level: * p < 0.1, ** p < 0.05, *** p < 0.01.

## C. Application Survey Weights

To check the impact of the composition of our sample on the results, we also estimate the models with survey weights. The weights are based on data on the population from the Federal Statistical Office of Germany (see Table 1). The data cover the population totals by gender, type of professorship, group of academic disciplines, institutional type, and geographic location, as defined by our control variables (see “Variables” section). We construct the weights using the raking-ratio method implemented in Stata. Applying the survey weights to the sample does not alter our findings in any meaningful way (see Appendix Table C.1).

**Table C.1:** Main treatment effects with survey weights applied to the sample

|  | **Full sample** | | **Sample without imputation** | | | |
| --- | --- | --- | --- | --- | --- | --- |
|  |  |  | Unweighted | | Weighted | |
|  | (1) | | (2) | | (3) | |
| **Panel A: General support** |  |  |  |  |  |  |
| Legal duty | 0.005 |  | 0.016 |  | 0.018 |  |
|  | (0.021) |  | (0.021) |  | (0.022) |  |
| Expectations public | -0.039 | * | -0.033 |  | -0.032 |  |
|  | (0.021) |  | (0.022) |  | (0.023) |  |
| Support public | 0.017 |  | 0.021 |  | 0.026 |  |
|  | (0.020) |  | (0.021) |  | (0.021) |  |
| Examples risks | -0.067 | *** | -0.071 | *** | -0.071 | *** |
|  | (0.022) |  | (0.023) |  | (0.023) |  |
| Controls | x |  | x |  | x |  |
| Observations | 4,091 |  | 3,797 |  | 3,797 |  |
| R-squared | 0.061 |  | 0.061 |  | 0.066 |  |
| **Panel B: Strong support** |  |  |  |  |  |  |
| Legal duty | -0.066 | *** | -0.072 | *** | -0.067 | *** |
|  | (0.019) |  | (0.020) |  | (0.020) |  |
| Expectations public | -0.062 | *** | -0.063 | *** | -0.066 | *** |
|  | (0.020) |  | (0.020) |  | (0.020) |  |
| Support public | -0.025 |  | -0.032 |  | -0.031 |  |
|  | (0.020) |  | (0.021) |  | (0.021) |  |
| Examples risks | -0.035 | * | -0.039 | * | -0.036 | * |
|  | (0.020) |  | (0.021) |  | (0.021) |  |
| Controls | x |  | x |  | x |  |
| Observations | 4,091 |  | 3,797 |  | 3,797 |  |
| R-squared | 0.058 |  | 0.058 |  | 0.058 |  |

*Notes:* The table shows the coefficients and standard errors of the treatment indicators estimated from linear probability models (ordinary least-squares regressions with robust standard errors) regressing the dummy variable indicating respondents’ (strong) support for an increase in engagement on the treatment indicators. Full sets of demographic, occupational, and engagement characteristics are always included. See “Variables” section and Appendix Table B.1 for details. While column (1) provides the baseline results for comparison, column (2) reports results for the restricted sample without missing values in any of the covariates that are used for weighting the sample. Column (3) uses population weights, which are based on gender, type of professorship, group of academic disciplines, institutional type, and geographic location, to weight each observation. The population totals are listed in Table 1 (population totals for the groups of academic disciplines were scaled to equal the totals of the other characteristics). Missing values for the control variables are imputed, and imputation dummies are included in the regressions; this excludes the variables used for the survey weights in columns (2) and (3). Significance level: * p < 0.1, ** p < 0.05, *** p < 0.01.

## D. Further Analyses: Effect Mechanism

As a complement to our main analysis of the mechanism underlying the treatment effects, we estimate the linear probability models of the main analysis separately for subgroups formed by splitting the sample based on the respondents’ elicitation estimates. One group comprises the respondents whose estimates correspond to the true value or are at least close to it. These respondents are compared to those who either under- or overestimate the true value (for details of the group assignment, see Appendix Table D.1). Three conditions should be fulfilled to conclude that the respondents’ prior level of knowledge is relevant for the treatment effects. First, there should be no treatment effects for those respondents whose elicitation estimates are (almost) correct. Second, we should observe treatment effects for those respondents who under- or overestimated the true value, at least in those cases where we observed treatment effects in the main analysis. Third, the direction of the treatment effects should differ between these two groups.

Focusing on the two cases where we observed significant treatment effects in the main analysis, the three conditions are only partly fulfilled (see Appendix Table D.1). The effect of the *Legal duty* treatment on strong support is indeed comparatively strong and highly significant for those underestimating the true value but still markedly negative and at least marginally significant among those whose estimates are (almost) correct. In the case of the *Expectations public* treatment and strong support, the respondents overestimating the true value show a particularly strong and significant treatment effect. However, the coefficient of the treatment indicator is negative for the other two groups as well, even though it is not significant. In the case of the *Support public* treatment, where we did not observe an effect in the main analysis, it is actually those whose estimates are (almost) correct who show a significant effect of the treatment on their support in general. Overall, we conclude that the evidence is too weak to argue that the respondents’ prior level of knowledge is systematically related to the treatment effects.

**Table D.1:** Effects of asymmetric respondents’ estimates of treatment information

|  | **General support** | | | | | |  | **Strong support** | | | | | |
| --- | --- | --- | --- | --- | --- | --- | --- | --- | --- | --- | --- | --- | --- |
|  | *Under* | | *Correct* | | *Over* | |  | *Under* | | *Correct* | | *Over* | |
|  | (1) | | (2) | | (3) | |  | (4) | | (5) | | (6) | |
| **Legal duty** | 0.005 |  | -0.010 |  | - |  |  | -0.078 | *** | -0.055 | * | - |  |
|  | (0.030) |  | (0.033) |  |  |  |  | (0.027) |  | (0.031) |  |  |  |
| Controls | x |  | x |  | x |  |  | x |  | x |  | x |  |
| Observations | 872 |  | 659 |  | - |  |  | 872 |  | 659 |  | - |  |
| R-squared | 0.068 |  | 0.121 |  |  |  |  | 0.088 |  | 0.097 |  |  |  |
| Control mean | 0.758 |  | 0.763 |  |  |  |  | 0.248 |  | 0.222 |  |  |  |
| **Expectations public** | -0.052 |  | -0.034 |  | -0.040 |  |  | -0.045 |  | -0.055 |  | -0.088 | ** |
|  | (0.035) |  | (0.036) |  | (0.048) |  |  | (0.032) |  | (0.034) |  | (0.044) |  |
| Controls | x |  | x |  | x |  |  | x |  | x |  | x |  |
| Observations | 641 |  | 561 |  | 362 |  |  | 641 |  | 561 |  | 362 |  |
| R-squared | 0.091 |  | 0.089 |  | 0.166 |  |  | 0.081 |  | 0.113 |  | 0.159 |  |
| Control mean | 0.750 |  | 0.780 |  | 0.730 |  |  | 0.208 |  | 0.255 |  | 0.260 |  |
| **Support public** | -0.032 |  | 0.075 | ** | -0.010 |  |  | -0.033 |  | -0.018 |  | 0.054 |  |
|  | (0.030) |  | (0.031) |  | (0.070) |  |  | (0.029) |  | (0.032) |  | (0.074) |  |
| Controls | x |  | x |  | x |  |  | x |  | x |  | x |  |
| Observations | 817 |  | 661 |  | 160 |  |  | 817 |  | 661 |  | 160 |  |
| R-squared | 0.088 |  | 0.121 |  | 0.169 |  |  | 0.086 |  | 0.099 |  | 0.226 |  |
| Control mean | 0.755 |  | 0.744 |  | 0.788 |  |  | 0.238 |  | 0.220 |  | 0.300 |  |

*Notes:* The table shows the coefficients and standard errors of the treatment indicators estimated from linear probability models (ordinary least-squares regressions with robust standard errors) regressing the dummy variable indicating respondents’ (strong) support for an increase in engagement on the treatment indicator for subgroups based on respondents’ elicitation estimates within the subsample consisting of the control group and the respective treatment group. Full sets of demographic, occupational, and engagement characteristics are always included. See “Variables” section and Appendix Table B.1 for details. The table also shows summary statistics of the model estimation and the mean of the dummy variable indicating respondents’ (strong) support for an increase in engagement in the control group. Subgroups ‘legal duty’ (true value = 15): “under” = 0-13, “correct” = 14-16; subgroups ‘expectations public’ (true value = 67): “under” = 0-59, “correct” = 60-70, “over” = 71-100; subgroups ‘support public’ (true value = 75): “under” = 0-69, “correct” = 70-80, “over” = 81-100. Missing values for the control variables are imputed, and imputation dummies are included in the regressions. Significance level: * p < 0.1, ** p < 0.05, *** p < 0.01.

## E. Robustness Check: Analysis Effect Mechanism

The analysis of whether an alteration of respondents’ prior knowledge or an increased salience of the frame provided is the mechanism responsible for the treatment effects observed is complicated by the fact that some respondents apparently corrected their initial estimates after having learned the true value from the treatment. Due to the structure and requirements of the survey in which the experiment was embedded, the questions eliciting respondents’ prior knowledge were located in the same section as the treatments. We check whether the resulting opportunity for correcting the initial estimates has been seized in two ways. First, we compare the shares of respondents who estimated exactly the true value between the treatment group to which the true value was revealed and the remainder of the sample. Second, we regress the absolute value of the deviation of respondents’ estimate from the true value on the indicator for the treatment group to which the true value was revealed. The results suggest that the initial estimates were indeed corrected in some cases.

The extent of corrections of the initial elicitation estimates is particularly pronounced in the case of the *Legal duty* treatment. The share of respondents estimating the correct value of federal states that implemented knowledge and technology transfer in their higher education laws is markedly higher in the treatment group to which the true value was revealed (29.0 percent) than in the remainder of the sample (4.1 percent). The regression shows that the estimates in that treatment group are on average 1.6 units closer to the true value of 15 than in the remainder of the sample and that this difference is highly significant (see Appendix Table E.1). This is a sizeable difference in the light of an average deviation in the remainder of the sample of 5.4 units. One explanation for the extent of corrections could be that respondents encountered comparatively greater difficulties with this elicitation question. This is at least suggested by the number of 265 missing responses, which is about twice as high as the number of missing responses for the two other elicitation questions (156 and 135).

The extent of corrections is less pronounced in the case of the *Expectations public* treatment, but still visible. The share of respondents estimating the correct value of the share of respondents to the public opinion poll who expect science to contribute to societal development is higher in the treatment group to which the true value was revealed (4.4 percent) than in the remainder of the sample (0.1 percent). The regression shows that the estimates in that treatment group are on average 1.5 units closer to the true value of 67 than in the remainder of the sample and that this difference is statistically significant (see Appendix Table E.1). However, this difference is put into perspective by an average deviation in the remainder of the sample of 17.7 units.

The extent of corrections is negligible in the case of the *Support public* treatment. The share of respondents estimating the correct value of the share of respondents to the public opinion poll who support academics’ engagement in public discussions is still higher in the treatment group to which the true value was revealed (8.8 percent) than in the remainder of the sample (5.8 percent). The regression shows that the estimates in that treatment group are on average 1.0 units closer to the true value of 75 than in the remainder of the sample, but this effect is not statistically significant (see Appendix Table E.1). Moreover, this difference is small compared to an average deviation in the remainder of the sample of 18.9 units.

The results of a robustness check confirm that respondents’ retrospective corrections do not distort our analysis of the effect mechanism. For this check, we estimate the linear probability models including a variable based on the elicitation estimates and an interaction term between this variable and the treatment indicators of the original analysis, but exclude all observations where the elicitation estimate corresponds exactly to the true value of the information provided by the treatment. This omission strongly reduces the extent to which the average deviation of the elicitation estimates from the true value differs between the treatment group to which the true value was revealed and the remainder of the sample: for the number of federal states, from -1.6 to -0.3, and for the shares of respondents to the public opinion poll, from -1.5 to -0.8 and from -1.0 to -0.4, respectively. As shown by the results in Appendix Table E.2, adapting the sample in this way does not lead to relevant differences with the original analysis. The only change concerning the interaction terms, which are of interest in this analysis, is that the marginal interaction term including the *Support public* treatment in the case of support in general becomes significant at the five percent level. We can thus preclude that the retrospective corrections obfuscate our analysis of the effect mechanism.

**Table E.1:** Differences in the accuracy of the elicitation estimates among experimental groups

|  | **Deviation estimates** | |
| --- | --- | --- |
|  | (1) | |
| **Legal duty** | -1.636 | *** |
|  | (0.178) |  |
| Constant | 5.436 | *** |
|  | (0.081) |  |
| Observations | 3,811 |  |
| R-squared | 0.021 |  |
|  |  |  |
| **Expectations public** | -1.488 | ** |
|  | (0.578) |  |
| Constant | 17.719 | *** |
|  | (0.260) |  |
| Observations | 3,932 |  |
| R-squared | 0.002 |  |
|  |  |  |
| **Support public** | -0.961 |  |
|  | (0.665) |  |
| Constant | 18.937 | *** |
|  | (0.308) |  |
| Observations | 3,954 |  |
| R-squared | 0.001 |  |

*Notes:* The table shows the results of ordinary least-squares regressions (with robust standard errors) of the deviation of the elicitation estimate (i.e., the absolute value of the difference between respondents’ estimate and the true value) on the treatment indicator for the full sample. The coefficients represent the average change in the deviation of the elicitation estimate from the true value due to belonging to the treatment group to which the true value was revealed; the constant represents the mean deviation within the remainder of the sample. Significance level: * p < 0.1, ** p < 0.05, *** p < 0.01.

**Table E.2:** Robustness check of the effects of average respondents’ estimates of treatment information

|  | **General support** | | | | | | | |  | **Strong support** | | | | | | | |
| --- | --- | --- | --- | --- | --- | --- | --- | --- | --- | --- | --- | --- | --- | --- | --- | --- | --- |
|  | *Full sample* | | | | *Reduced sample* | | | |  | *Full sample* | | | | *Reduced sample* | | | |
|  | (1) | | (2) | | (3) | | (4) | |  | (5) | | (6) | | (7) | | (8) | |
| Legal duty | 0.004 |  | 0.003 |  | 0.008 |  | 0.025 |  |  | -0.068 | *** | -0.059 | ** | -0.055 | ** | -0.029 |  |
|  | (0.021) |  | (0.025) |  | (0.023) |  | (0.032) |  |  | (0.019) |  | (0.024) |  | (0.022) |  | (0.031) |  |
| Legal duty difference |  |  | -0.001 |  |  |  | 0.000 |  |  |  |  | 0.004 | ** |  |  | 0.005 | ** |
|  |  |  | (0.002) |  |  |  | (0.002) |  |  |  |  | (0.002) |  |  |  | (0.002) |  |
| Legal duty x  legal duty difference |  |  | 0.000 |  |  |  | -0.003 |  |  |  |  | -0.001 |  |  |  | -0.005 |  |
|  |  |  | (0.004) |  |  |  | (0.005) |  |  |  |  | (0.004) |  |  |  | (0.004) |  |
| Expectations public | -0.041 | * | -0.038 |  | -0.048 | ** | -0.036 |  |  | -0.063 | *** | -0.092 | *** | -0.072 | *** | -0.095 | *** |
|  | (0.021) |  | (0.029) |  | (0.023) |  | (0.031) |  |  | (0.020) |  | (0.026) |  | (0.021) |  | (0.028) |  |
| Expectations public difference |  |  | -0.001 | * |  |  | -0.001 |  |  |  |  | 0.000 |  |  |  | 0.000 |  |
|  |  |  | (0.001) |  |  |  | (0.001) |  |  |  |  | (0.001) |  |  |  | (0.001) |  |
| Expectations public x  expectations public difference |  |  | 0.000 |  |  |  | -0.001 |  |  |  |  | 0.002 |  |  |  | 0.001 |  |
|  |  |  | (0.001) |  |  |  | (0.001) |  |  |  |  | (0.001) |  |  |  | (0.001) |  |
| Support public | 0.015 |  | 0.044 | * | 0.019 |  | 0.063 | ** |  | -0.026 |  | -0.025 |  | -0.027 |  | -0.021 |  |
|  | (0.020) |  | (0.026) |  | (0.021) |  | (0.028) |  |  | (0.020) |  | (0.025) |  | (0.021) |  | (0.028) |  |
| Support public difference |  |  | -0.001 | * |  |  | -0.001 |  |  |  |  | 0.000 |  |  |  | 0.001 |  |
|  |  |  | (0.001) |  |  |  | (0.001) |  |  |  |  | (0.001) |  |  |  | (0.001) |  |
| Support public x  support public difference |  |  | -0.002 | * |  |  | -0.002 | ** |  |  |  | 0.000 |  |  |  | 0.000 |  |
|  |  |  | (0.001) |  |  |  | (0.001) |  |  |  |  | (0.001) |  |  |  | (0.001) |  |
| Controls | x | | x | | x | | x | |  | x | | x | | x | | x | |
| Observations | 3,305 |  | 3,305 |  | 2,773 |  | 2,773 |  |  | 3,305 |  | 3,305 |  | 2,773 |  | 2,773 |  |
| R-squared | 0.064 |  | 0.071 |  | 0.064 |  | 0.072 |  |  | 0.065 |  | 0.069 |  | 0.069 |  | 0.074 |  |

*Notes:* The table shows the coefficients and standard errors of the treatment indicators and, in columns (2), (4), (6) and (8), of the variables consisting of the absolute value of the difference between respondents’ elicitation estimates of the information provided by the treatments and its true value and the interaction term between these two variables estimated from linear probability models (ordinary least-squares regressions with robust standard errors) regressing the dummy variable indicating respondents’ (strong) support for an increase in engagement on the variables and the interaction terms indicated for the subsample consisting of the control group and the treatment groups listed. Columns (3), (4), (7) and (8) run the regressions on a reduced sample that drops all observations where an estimate of any of the elicitation questions is equal to the true value. Columns (1), (2), (5) and (6) provide the results on the full sample for comparison. Full sets of demographic, occupational, and engagement characteristics are always included. See “Variables” section and Appendix Table B.1 for details. Missing values for the control variables and the elicitation estimates are imputed, and imputation dummies are included in the regressions. Significance level: * p < 0.1, ** p < 0.05, *** p < 0.01.
